# COVID-19-related school closures and students’ achievement and motivation: Did teacher support make a difference

**DOI:** 10.1038/s41539-026-00439-1

**Published:** 2026-08-19

**Authors:** Jessika Golle, Patrick Lösche, Hanna Beißert, Ann-Kathrin Wieland, Regina Rieber, Johannes Schult, Marcus Hasselhorn, Benjamin Nagengast, Ulrich Trautwein

**Affiliations:** 1 Hector Research Institute of Education Sciences and Psychology, University of Tübingen, Tübingen, Germany; 2 DIPF|Leibniz Institute for Research and Information in Education, Frankfurt am Main, Germany; 3 Department of Educational Sciences, Goethe-University Frankfurt am Main, Frankfurt am Main, Germany; 4 Institute for Educational Analyses Baden-Württemberg, Stuttgart, Germany; 5 MTO Psychological Research and Consulting GmbH, Tübingen, Germany

**Keywords:** Scientific community, Education, Social sciences, Education

## Abstract

Most effects of the COVID-19 pandemic on student outcomes were negative, though school systems varied in their ability to mitigate learning losses. In this study, we examined teacher–student contact as a key factor for buffering pandemic-related risks to achievement and motivation. We analyzed multicohort data collected at the beginning and end of the school year from 2740 first-grade students in Baden-Württemberg, Germany. Against expectations, no overall decline in achievement emerged under pandemic schooling; however, high teacher–student contact was associated with higher math achievement compared with regular schooling. Motivational outcomes showed negative average effects for interest and self-concept in language domains, particularly under low teacher–student contact and low family socioeconomic status. Owing to its timing at the start of formal schooling during the first wave of the pandemic, this study provides unique evidence of the role of teacher–student contact in students’ achievement and motivation during crisis conditions.

## Introduction

Although the overall pattern of findings has supported a substantial negative effect of the COVID-19 pandemic on achievement and, somewhat less clearly, on student motivation, not all classes and students (even within the same school districts) were equally affected by these school closings. The most recent meta-analyses reported an overall loss in school achievement of *d* = −0.21^1^, *d* = −0.14^2^, and *d* = −0.19^3^ due to distance learning during the pandemic (independent of the dates of the school closures or students’ grade levels) and a remarkable degree of variation in effect sizes across studies, ranging from *d* = −0.65 to *d* = +0.07^2^. Losses have varied on the basis of a country’s income (linked to the duration of the school closures and the availability of resources to facilitate remote learning), students’ family backgrounds, and the subject that was considered^4–8^. In contrast to studies that investigated losses in students’ achievement, the number of empirical studies that considered potential losses in motivation is still small, and there are no systematic reviews or meta-analyses that have presented systematic effects of schooling during the pandemic on students’ motivation. However, the evidence points to negative effects on motivation in college and university students^9–12^ as well as in primary and secondary school students^13,14^. Various student characteristics have been discussed as potential moderators of effects, such as self-control^10^, family background, gender, and special educational needs^13,15^. This small number of studies on how the pandemic affected student motivation is remarkable given the importance of student motivation as a key outcome of education in itself and because motivation has a long-term effect on other central outcomes, such as persistence, achievement, and academic choices^16^.

The lack of homogeneity observed in effects was shown to be associated with the subject that was considered, students’ family background, or initial student achievement^4,17^. However, from a psychological point of view, there has been a crucial shortcoming so far: the lack of attention to key constructs from educational psychology that might explain between-classroom and within-classroom differences in effects of the COVID-19 pandemic on achievement and motivation. In fact, to explain heterogeneity in the findings, several variables have been discussed, such as the amount of time students spent on schoolwork and teachers’ and students’ familiarity with online learning resources^2,7,18^. However, these constructs have not been used in empirical studies, most likely due to limitations in the respective data sets.

In this study, we focused on the frequency of teacher-student interactions as an aspect of (individual) student support. Student support is a key variable in educational psychology and is part of leading teaching-quality frameworks, such as the Pianta and Hamre classroom process model^19^ and the framework of three basic dimensions of teaching quality^20,21^. At its core, student support means that teachers are aware of individual students’ needs for support in terms of understanding and motivation and that teachers are willing and capable of providing appropriate support on time. It is evident that the school closures during the pandemic made it challenging for teachers to provide an adequate level of support to their students. Among other actions, teachers tried to overcome these challenges by making phone calls to parents and students, being available for online sessions, delivering learning materials to students’ homes, and grading and commenting on students’ work. However, on average, according to teacher and parent reports, individual contact between teachers and students was rare, and there was a lot of variation between teachers^22,23^. Thus, we expected to find average negative effects on achievement and motivation due to the pandemic caused by reductions and impairments in teacher support^24–26^. Thereby, teacher support should be particularly relevant for students’ interest or intrinsic value related to a specific subject^27^. In addition, we expected to find large differences between teachers in how much support they provided to their classes and differences in how much attention individual students received, resulting in larger negative effects for students with less contact with their teachers.

In this study, we examined the effects of the COVID-19-related school closures in the first five months of the pandemic on students’ achievement and motivation in a sample of first-grade students in the federal state of Baden-Württemberg in Germany (at the beginning of compulsory schooling). For detailed information about the situation in Germany and especially in Baden–Württemberg during the first lockdown, see Notes S1 and S2. We conducted a detailed examination of teacher-student contact, which might have exacerbated or prevented negative effects of the school closures. To this end, we used data from a longitudinal multicohort study in which Cohort 1 had attended first grade before the pandemic (September 2018 to June 2019), whereas students in Cohort 2 had been affected by the pandemic during first grade (September 2019 to June 2020). Subject-specific school achievement and motivation were assessed at the beginning and end of Grade 1, and, in Cohort 2, additional measures were administered to capture the circumstances under which the students were learning during the school closures. By exploring these circumstances, we were able to examine psychologically relevant processes. Furthermore, we were able to control for students’ characteristics before the pandemic as well as class- and school-level variables. Thus, we were able to control for potential differences between the two cohorts before the pandemic occurred. We used class size and proportion of students with a migration background on the school level because we assumed that these variables might have affected teaching under pandemic conditions^28,29^.

We investigated the following research questions:

(RQ 1) To what extent was Grade 1 students’ achievement impaired by the pandemic, and was this effect moderated by students’ family SES or prior achievement?

(RQ 2) To what extent was Grade 1 students’ motivation impaired by the pandemic, and was this effect moderated by students’ family SES or prior motivation?

(RQ 3) Did students with low, medium, or high teacher-student contact during the pandemic-related school closures show different levels of achievement and motivation compared with students before the pandemic, and were these effects moderated by students‘ family SES, or prior achievement, or prior motivation?

## Results

Tables 1–6 provide an overview of the sample, all measures used in this study, and the descriptive statistics.

**Table 1:** Overview of sample sizes

Number of	Total	Written consent	Cohort 1 at T1 and T2	Cohort 2 at T1	Cohort 2 at T2
Schools	163	163	88	94	61
Classes	182	182	88	94	61
Students	3451	2740	1405	1335	873
School principals^a^	-	163	88	75	48
Teachers	-	245	130	115	58
Parent reports^b^	-	2248	1157	1091	721

The complete sample comprised 3451 students; the mean class size was 19 students (ranging from 4 to 30). But there was no written informed consent from 711 parents at either measurement occasion. On average, 80% of the parents in a class agreed that their child could participate in the study (written informed consent per class ranged from 18% to 100%).

^a^ School principals were asked once to fill out a questionnaire.

^b^ Parent questionnaires could have been answered by a child’s parents but also a child’s other relatives, but we call them parent reports for simplicity.

**Table 2:** Sample characteristics and official statistics

Data sources	Number of students in a class			% Girls	% Students who do not speak German with their parents	% Migration background^e^	HISEI	
	*M*	Min	Max				*M*	SD
Official statistics^a^								
2018/2019	19.80	-	-	49.19	-	30.38	-	-
2019/2020	19.80	-	-	48.90	^-^	30.53	-	-
VERA 2021^b^	-	-	-	49.40	22.50	-	-	-
VERA 2022^c^	-	-	-	49.20	24.00	-	-	-
IQB 2021^d^	-	-	-	-	-	-	51.50	21.10
Study sample								
Cohort 1	20.05	7	29	49.00	16.96	27.13	58.55	19.50
Cohort 2a	17.96	4	30	50.26	21.38	25.47	57.84	19.37
Cohort 2b	19.96	6	30	50.06	21.77	23.44	58.26	19.28
Both cohorts	18.97	4	30	49.60	19.11	26.34	58.21	19.44

To evaluate the representativeness of our sample, we compared our sample with official statistics from the state education ministry and monitoring results on the state level (VERA 2021, VERA 2022) as well as for Germany (IQB 2021) for both cohorts if available. Cohort 2a indicates the whole Cohort 2 starting in September 2019. Cohort 2b indicates schools that remained in the sample despite the pandemic. The number of students in a class represents the actual numbers of students per class based on teacher reports.

^a^The reference values are from public primary schools in Baden-Württemberg. No detailed information is available about class sizes for Grade 1.

^b^We used VERA 3 data from 2021 (https://web.archive.org/web/20250517223359/https://ibbw-bw.de/site/pbs-bw-km-root/get/documents_E-1988448993/KULTUS.Dachmandant/KULTUS/Dienststellen/ibbw/Systemanalysen/Bildungsberichterstattung/Ergebnisberichte/VERA_3/Ergebnisse_VERA3_2021.pdf). This cohort is equivalent to Cohort 1 in this study because data were collected on all third graders in Baden-Württemberg.

^c^We used VERA 3 data from 2022 (https://web.archive.org/web/20240407001117/https://ibbw-bw.de/site/pbs-bw-km-root/get/documents_E-1295347296/KULTUS.Dachmandant/KULTUS/Dienststellen/ibbw/Systemanalysen/Bildungsberichterstattung/Ergebnisberichte/VERA_3/Ergebnisse_VERA3_2022.pdf). This cohort is equivalent to Cohort 2 in this study because data were collected from all third graders in Baden-Württemberg.

^d^ We used IQB Bildungstrend 2021 data to assess the representativeness of our sample with respect to SES because the state ministry provided no data (https://www.iqb.hu-berlin.de/media/documents/IQB_Bildungstrend2021_Berichtsband.pdf). However, caution is necessary when interpreting the monitoring data because it is a different cohort of students. In IQB Bildungstrend 2021, the data came from fourth-grade students.

^e^On the state level, foreign citizens without German citizenship and German citizens with a migration background were considered together. In our sample, a migration background was defined by the mother, father, or child being born outside Germany (e.g., even if only one child was born outside of Germany and both parents were born in Germany).

**Table 3:** Descriptive statistics (pretest measurement)

Variables		Entire sample (*N* = 2740)					Cohort 1 (*N* = 1405)				Cohort 2^a^ (*N* = 1335)				Baseline differences^b^	
	*M*	SD	Range		Rate of missing data	a	*M*	SD	Range		*M*	SD	Range			
			Min	Max					Min	Max			Min	Max	ES in *SD*_*Y*_	*p-*value
Individual level																
Demographics																
Age	6.58	0.39	5.33	9.12	0.01		6.56	0.37	5.7	8.72	6.60	0.41	5.33	9.12	0.11	0.026
Girls	0.50	0.50	0	1	0.00		0.49	0.50	0	1	0.50	0.50	0	1	0.03	0.468
Migration	0.26	0.44	0	1	0.30		0.27	0.44	0	1	0.25	0.44	0	1	−0.05	0.578
HISEI	58.21	19.44	11.56	88.96	0.23		58.55	19.50	11.74	88.96	57.84	19.37	11.56	88.96	−0.04	0.478
Achievement																
Reading	20.90	27.51	0	155	0.01	0.98	19.83	25.97	0	154	22.01	29	0	155	0.08	0.058
Mathematics	26.93	7.93	0	53	0.01	0.92	26.98	7.93	2	53	26.87	7.94	0	51	−0.01	0.799
Cognitive ability	8.74	4.22	0	25	0.03	0.88	8.64	4.18	0	25	8.85	4.27	0	23	0.05	0.227
Motivation																
Mathematics	2.53	0.35	1	3	0.03	0.57	2.53	0.36	1	3	2.52	0.35	1	3	−0.03	0.483
Language	2.41	0.38	1	3	0.02	0.54	2.38	0.38	1.17	3	2.43	0.38	1	3	0.13	0.004
Class level																
Students per class	18.96	5.52	4	30	0.00		20.02	4.71	7	29	17.96	6.04	4	30	-0.28	0.048
School level																
% of students migration background	25.11	20.08	0	87	0.26		24.02	19.68	0	80	25.94	20.47	1	87	0.02	0.897

To test whether baseline differences existed, we ran a regression model in which all baseline measures were predicted by the cohort variable. The results are presented in the column labeled *Baseline differences*. For the analysis, we used Mplus 8.5^59^. To account for potentially nonnormally distributed variables and the multilevel structure of the data (students nested in classes), we used cluster-robust standard errors^60^. To handle missing values, we used full information maximum likelihood (FIML) estimation.

^a^ Total Cohort 2 sample.

^b^ Baseline differences between Cohort 1 and Cohort 2.

**Table 4:** Descriptive statistics (pretest measurement)

Variables		Entire sample (*N* = 2740)					Cohort 2^a^ (*N* = 1335)				Cohort 2^b^ (*N* = 873)				Drop-out prediction in Cohort 2
	*M*	SD	Range		Miss	a	*M*	SD	Range		*M*	SD	Range		
			Min	Max					Min	Max			Min	Max	*OR* [95% CI]
Individual level															
Demographics															
Age	6.58	0.39	5.33	9.12	0.01		6.60	0.41	5.33	9.12	6.61	0.41	5.8	9.12	[0.551, 1.160]
Girls	0.50	0.50	0	1	0.00		0.50	0.50	0	1	0.50	0.50	0	1	[0.829, 1.290]
Migration	0.26	0.44	0	1	0.30		0.25	0.44	0	1	0.23	0.42	0	1	[0.807, 1.933]
HISEI	58.21	19.44	11.56	88.96	0.23		57.84	19.37	11.56	88.96	58.26	19.28	14.21	88.96	[0.988, 1.005]
Achievement															
Reading	20.90	27.51	0	155	0.01	0.98	22.01	29.00	0	155	21.63	28.05	0	155	[0.995, 1.006]
Mathematics	26.93	7.93	0	53	0.01	0.92	26.87	7.94	0	51	26.85	7.71	2	51	[0.984, 1.031]
Cognitive ability	8.74	4.22	0	25	0.03	0.88	8.85	4.27	0	23	8.91	4.28	0	23	[0.954, 1.016]
Motivation															
Mathematics	2.53	0.35	1	3	0.03	0.57	2.52	0.35	1	3	2.51	0.36	1	3	[0.863, 1.934]
Language	2.41	0.38	1	3	0.02	0.54	2.43	0.38	1	3	2.42	0.39	1	3	[0.759, 1.881]
Class level															
Students per class	18.96	5.52	4	30	0.00		17.96	6.04	4	30	18.41	6.26	6	30	[0.882, 1.014]
School level															
% of students migration background	25.11	20.08	0	87	0.26		25.94	20.47	1	87	24.93	19.87	1	75	[0.981, 1.032]

To test whether drop-out in Cohort 2 could be predicted by any pretest measure, we ran a logistic regression model. Findings are presented in the column labeled *Drop-out prediction in Cohort 2*. For the analysis, we used Mplus 8.5^59^. To account for potentially nonnormally distributed variables and the multilevel structure of the data (students nested in classes), we used cluster-robust standard errors^60^. To handle missing values, we used full information maximum likelihood (FIML) estimation.

^a^ Total Cohort 2 sample.

^b^ Participants from Cohort 2 who participated at both T1 and T2.

**Table 5:** Descriptive statistics (posttest measurement)

Variables		Entire sample (*N* = 2740)					Cohort 1 (*N* = 1405)				Cohort 2 (*N* = 873)			
	*M*	SD	Range		Rate of missing data	a	*M*	SD	Range		*M*	SD	Range	
			Min	Max					Min	Max			Min	Max
Achievement														
Reading	131.89	27.39	2	159	0.22	0.95	131.66	26.1	6	159	132.28	29.47	2	159
Mathematics	40.22	6.58	0	53	0.22	0.91	40.28	6.61	0	53	40.12	6.52	13	53
Motivational variables														
Intrinsic value														
Mathematics	2.52	0.56	1	3	0.16	0.86	2.52	0.58	1	3	2.53	0.53	1	3
Reading	2.64	0.53	1	3	0.16	0.88	2.65	0.52	1	3	2.61	0.54	1	3
Writing	2.56	0.54	1	3	0.16	0.88	2.59	0.54	1	3	2.52	0.55	1	3
Self-concept														
Mathematics	2.60	0.49	1	3	0.16	0.85	2.60	0.50	1	3	2.60	0.48	1	3
Reading	2.67	0.46	1	3	0.17	0.84	2.69	0.44	1	3	2.65	0.48	1	3
Writing	2.64	0.45	1	3	0.16	0.81	2.67	0.44	1	3	2.60	0.46	1	3
Treatment moderator—original scale														
Contact	-	-	-	-	-	-	-	-	-	-	3.03	0.89	1	5
Treatment moderator—low, medium, high														
Contact	-	-	-	-	-	-	-	-	-	-	0.98	0.72	0	2

In Cohort 1, the rate of missing values ranged from 0.04 to 0.07. In Cohort 2, the rate of missing values ranged from 0.26 to 0.41 because 33 schools decided not to participate in this study at the end of Grade 1 due to the pandemic.

**Table 6:** Correlations

		1	2	3	4	5	6	7	8	9	10	11	12	13	14	15	16	17	18	19	20	21	22
Individual level																							
Demographics																							
Age	1	1.00	−0.06	0.06	−0.13	0.11	0.06	0.05	0.04	0.01	0.02	0.05	0.02	−0.02	−0.03	−0.07	−0.03	0.02	−0.05	−0.02	0.01	−0.03	0.02
Female	2	−0.06	1.00	0.01	−0.02	0.05	−0.21	0.11	−0.08	0.12	0.00	0.01	−0.03	0.03	0.02	−0.01	−0.27	−0.07	0.13	0.10	−0.16	0.02	0.07
Migration	3	0.06	0.01	1.00	−0.32	−0.03	−0.16	−0.05	0.02	0.01	0.03	0.25	0.09	−0.09	−0.06	−0.12	−0.13	0.09	0.02	0.03	0.03	−0.01	−0.01
HISEI	4	−0.13	−0.02	−0.32	1.00	0.17	0.25	0.17	0.07	0.07	0.01	−0.13	−0.09	0.09	0.07	0.23	0.23	−0.07	0.09	−0.03	0.01	0.16	0.05
Achievement																							
Reading	5	0.11	0.05	−0.03	0.17	1.00	0.40	0.20	0.18	0.31	−0.04	0.02	−0.01	0.01	0.00	0.26	0.29	0.01	0.09	0.03	0.05	0.20	0.09
Mathematics	6	0.06	−0.21	−0.16	0.25	0.40	1.00	0.35	0.31	0.10	0.03	−0.08	−0.03	0.03	0.02	0.43	0.69	0.12	0.02	−0.06	0.24	0.19	0.02
Cognitive ability	7	0.05	0.11	−0.05	0.17	0.20	0.35	1.00	0.12	0.08	0.02	−0.04	0.01	−0.01	−0.01	0.22	0.28	0.04	0.00	−0.02	0.09	0.06	0.01
Motivation																							
Mathematics	8	0.04	−0.08	0.02	0.07	0.18	0.31	0.12	1.00	0.48	−0.01	−0.04	0.12	−0.12	−0.12	0.17	0.29	0.18	0.12	0.10	0.20	0.19	0.09
Language	9	0.01	0.12	0.01	0.07	0.31	0.10	0.08	0.48	1.00	−0.05	0.01	0.00	0.00	−0.02	0.09	0.06	0.08	0.16	0.12	0.07	0.18	0.12
Class Level																							
Students per class	10	0.02	0.00	0.03	0.01	−0.04	0.03	0.02	−0.01	−0.05	1.00	0.03	−0.01	0.01	0.01	−0.01	0.01	−0.03	−0.06	−0.03	−0.01	−0.04	0.00
School level																							
Percentage of students with migration background	11	0.05	0.01	0.25	−0.13	0.02	−0.08	−0.04	−0.04	0.01	0.03	1.00	−0.05	0.05	0.03	−0.02	−0.04	0.07	−0.01	0.04	0.01	0.01	0.00
Student−teacher contact																							
Contact teacher−students (original scale)	12	0.02	−0.03	0.09	−0.09	−0.01	−0.03	0.01	0.12	0.00	−0.01	−0.05	1.00	−1.00	−0.95	−0.16	−0.13	0.12	−0.02	0.00	0.06	−0.03	0.03
Contact teacher−students (recoded)	13	−0.02	0.03	−0.09	0.09	0.01	0.03	−0.01	−0.12	0.00	0.01	0.05	−1.00	1.00	0.95	0.16	0.13	−0.12	0.02	0.00	−0.06	0.03	−0.03
Contact teacher−students (0,1,2)	14	−0.03	0.02	−0.06	0.07	0.00	0.02	−0.01	−0.12	−0.02	0.01	0.03	−0.95	0.95	1.00	0.13	0.11	−0.11	0.01	0.00	−0.04	0.02	0.01
Outcomes																							
Achievement																							
Reading	15	−0.07	−0.01	−0.12	0.23	0.26	0.43	0.22	0.17	0.09	−0.01	−0.02	−0.16	0.16	0.13	1.00	0.50	−0.01	0.23	0.06	0.05	0.39	0.11
Mathematics	16	−0.03	−0.27	−0.13	0.23	0.29	0.69	0.28	0.29	0.06	0.01	−0.04	−0.13	0.13	0.11	0.50	1.00	0.16	0.02	−0.04	0.27	0.17	0.01
Motivational variables																							
Intrinsic value																							
Mathematics	17	0.02	−0.07	0.09	−0.07	0.01	0.12	0.04	0.18	0.08	−0.03	0.07	0.12	−0.12	−0.11	−0.01	0.16	1.00	0.16	0.29	0.68	0.08	0.15
Reading	18	−0.05	0.13	0.02	0.09	0.09	0.02	0.00	0.12	0.16	−0.06	−0.01	−0.02	0.02	0.01	0.23	0.02	0.16	1.00	0.46	0.08	0.60	0.29
Writing	19	−0.02	0.10	0.03	−0.03	0.03	−0.06	−0.02	0.10	0.12	−0.03	0.04	0.00	0.00	0.00	0.06	−0.04	0.29	0.46	1.00	0.17	0.27	0.59
Self−concept																							
Mathematics	20	0.01	−0.16	0.03	0.01	0.05	0.24	0.09	0.20	0.07	−0.01	0.01	0.06	−0.06	−0.04	0.05	0.27	0.68	0.08	0.17	1.00	0.17	0.21
Reading	21	−0.03	0.02	−0.01	0.16	0.20	0.19	0.06	0.19	0.18	−0.04	0.01	−0.03	0.03	0.02	0.39	0.17	0.08	0.60	0.27	0.17	1.00	0.37
Writing	22	0.02	0.07	−0.01	0.05	0.09	0.02	0.01	0.09	0.12	0.00	0.00	0.03	−0.03	0.01	0.11	0.01	0.15	0.29	0.59	0.21	0.37	1.00

The typical challenge of weaker students receiving more teacher support might not be problematic in this study because we did not observe any substantial correlation between baseline performance and teacher−student contact.

### Baseline equivalence

On the individual level, students in Cohort 2 were significantly older, ${b}_{{age}}=0.11,\,p=0.026$, more motivated with respect to language activities, $b_{{\rm{mot}}\, lang} = -0.28, p = 0.048$, and slightly better in terms of reading achievement, ${b}_{{read}}=0.08,\,p=0.058$, than the students in Cohort 1. The difference in age might be explained by the fact that a few students in Cohort 2 were older than those in Cohort 1 and by a difference in testing of approximately one week due to holidays, but this difference should not have affected the test scores. And there were smaller class sizes in Cohort 2 than in Cohort 1, ${b}_{{class\; size}}=-0.28,{p}=.048$. For all other pretest measures, there were no meaningful (i.e., statistically significant or ES in SD_Y_ > 0.05; see ref. ^30^) differences between the two cohorts at the beginning of Grade 1. For more details, see Table 3. To ensure that we controlled for all possible baseline differences in the observed variables in our sample, we included all measures that were assessed at the beginning of Grade 1 and were relevant for this study as control variables in our analyses.

### Main outcomes 1—effects on students’ achievement

Contrary to our expectations, the findings revealed no significant negative effects of schooling during the pandemic on students’ reading achievement, ${b}_{{read}}=0.03,\,p=0.718$, or mathematical achievement, ${b}_{{math}}=-0.01,{p}=.878$, at the end of Grade 1 compared with schooling under regular conditions (while controlling for any potential baseline differences between the two cohorts). See Table 7 for further details. To test whether students’ family SES or initial achievement moderated these effects, we built corresponding interactions with cohort. However, for reading achievement, we refrained from investigating any interaction effects due to the distribution of this variable in our sample (see Fig. S1)^31,32^. For mathematics at the end of Grade 1, we included the interactions between cohort and students’ family SES or cohort and baseline achievement in the models; but none of these interactions were statistically significant (all $p{\rm{s}}\ge 0.693)$. Although we had expected to find that students’ family SES or baseline performance moderated the effects of the pandemic on achievement, we found no empirical evidence to support these hypotheses. For further details, see Tables 8 and 9.

**Table 7:** Effects of the COVID-19 pandemic

Variables	Multiple regression coefficient (ES in *SD*_*Y*_)	95% CI		SE	*z*	*p*
		lower	upper			
Achievement						
Reading	0.03	−0.11	0.16	0.07	0.36	0.718
Mathematics	−0.01	−0.10	0.08	0.05	−0.15	0.878
Motivational variables						
Intrinsic value						
Mathematics	0.04	−0.06	0.13	0.05	0.75	0.456
Reading	−0.12	−0.22	-0.02	0.05	−2.42	0.016
Writing	−0.15	−0.24	-0.06	0.05	−3.37	0.001
Self-concept						
Mathematics	0.01	−0.08	0.10	0.05	0.28	0.781
Reading	−0.14	−0.24	−0.05	0.05	−2.97	0.003
Writing	−0.17	−0.26	−0.08	0.05	−3.68	<0.001

The table presents the regression coefficients for cohort (treatment variable) in a multiple linear regression analysis in which all dependent variables were considered simultaneously, and all pretest measures were used as control variables. We ran the analysis in Mplus 8.5^59^. To account for potentially nonnormally distributed variables and the multilevel structure of the data (students nested in classes), we used cluster-robust standard errors^60^. To handle missing values, we used full information maximum likelihood (FIML) estimation. All variables were z-standardized before the analysis except for the dichotomous variables (cohort, gender, and migration background). Thus, the regression coefficients can be interpreted as effect sizes.

**Table 8:** Effects of the COVID-19 pandemic—moderated by HISEI

Variables	Cohort effect (ES in *SD*_*Y*_)				HISEI (ES in *SD*_*Y*_)				Interaction term Cohort × HISEI (ES in *SD*_*Y*_)			
	b	SE	*z*	*p*	b	SE	*z*	*p*	b	SE	*z*	*p*
Achievement												
Reading	0.02	0.07	0.35	0.723	0.12	0.03	3.88	<0.001	-	-	-	-
Mathematics	−0.01	0.05	−0.16	0.877	0.04	0.02	1.76	0.078	<|0.01|	0.04	0.06	0.950
Motivational variables												
Intrinsic value												
Mathematics	0.04	0.05	0.74	0.461	−0.09	0.03	−2.77	0.006	0.01	0.04	0.28	0.779
Reading	−0.12	0.05	−2.47	0.014	0.06	0.03	1.69	0.091	0.09	0.05	1.81	0.071
Writing	−0.16	0.05	−3.39	0.001	−0.03	0.04	−0.84	0.398	0.03	0.05	0.66	0.510
Self-concept												
Mathematics	0.01	0.05	0.27	0.785	−0.06	0.04	−1.73	0.083	0.01	0.05	0.15	0.881
Reading	−0.15	0.05	−3.04	0.002	0.06	0.03	2.31	0.021	0.12	0.05	2.41	0.016
Writing	−0.17	0.05	−3.67	<0.001	0.05	0.03	1.69	0.092	−0.02	0.05	−0.34	0.732

The table presents the regression coefficients for cohort (treatment variable), HISEI score, and the interaction between cohort and HISEI score in a multiple linear regression analysis in which all dependent variables were considered simultaneously, and all pretest measures were used as control variables. We ran the analysis in Mplus 8.5^59^. To account for potentially nonnormally distributed variables and the multilevel structure of the data (students nested in classes), we used cluster-robust standard errors^60^. To handle missing values, we used full information maximum likelihood (FIML) estimation. All variables were z-standardized before the analysis except for the dichotomous variables (cohort, gender, and migration background). Thus, the regression coefficients can be interpreted as effect sizes.

**Table 9:** Effects of the COVID-19 pandemic—moderated by baseline level

Variables	Cohort effect (ES in *SD*_*Y*_)				Pretest (ES in *SD*_*Y*_)				Interaction term Cohort × Pretest (ES in *SD*_*Y*_)			
	b	SE	*z*	*p*	b	SE	*z*	*p*	b	SE	*z*	*p*
Achievement												
Reading	0.02	0.07	0.35	0.723	0.09	0.01	6.09	<0.001	-	-	-	-
Mathematics	−0.01	0.05	−0.19	0.851	0.59	0.03	20.5	<0.001	0.02	0.04	0.40	0.693
Motivational variables												
Intrinsic value												
Mathematics	0.03	0.05	0.69	0.491	0.19	0.04	5.02	<0.001	−0.11	0.05	−2.21	0.027
Reading	−0.13	0.05	−2.50	0.012	0.06	0.03	2.00	0.046	0.08	0.04	1.85	0.065
Writing	−0.15	0.05	−3.36	0.001	0.06	0.03	1.83	0.068	0.01	0.04	0.37	0.708
Self-concept												
Mathematics	0.01	0.05	0.17	0.868	0.13	0.04	3.51	<0.001	−0.06	0.04	−1.45	0.146
Reading	−0.15	0.05	−3.03	0.002	0.08	0.03	2.75	0.006	0.04	0.04	1.11	0.265
Writing	−0.17	0.05	−3.69	<0.001	0.06	0.03	1.92	0.055	0.03	0.04	0.73	0.468

The table presents the regression coefficients for cohort (treatment variable), pretest score, and the interaction term between cohort and pretest score in a multiple linear regression analysis in which all dependent variables were considered simultaneously, and all pretest measures were used as control variables. We ran the analysis in Mplus 8.5^59^. To account for potentially nonnormally distributed variables and the multilevel structure of the data (students nested in classes), we used cluster-robust standard errors^60^. To handle missing values, we used full information maximum likelihood (FIML) estimation. All variables were z-standardized before the analysis except for the dichotomous variables (cohort, gender, and migration background). Thus, the regression coefficients can be interpreted as effect sizes.

### Main outcomes 2—effects on students’ motivation

**Fig. 1:**
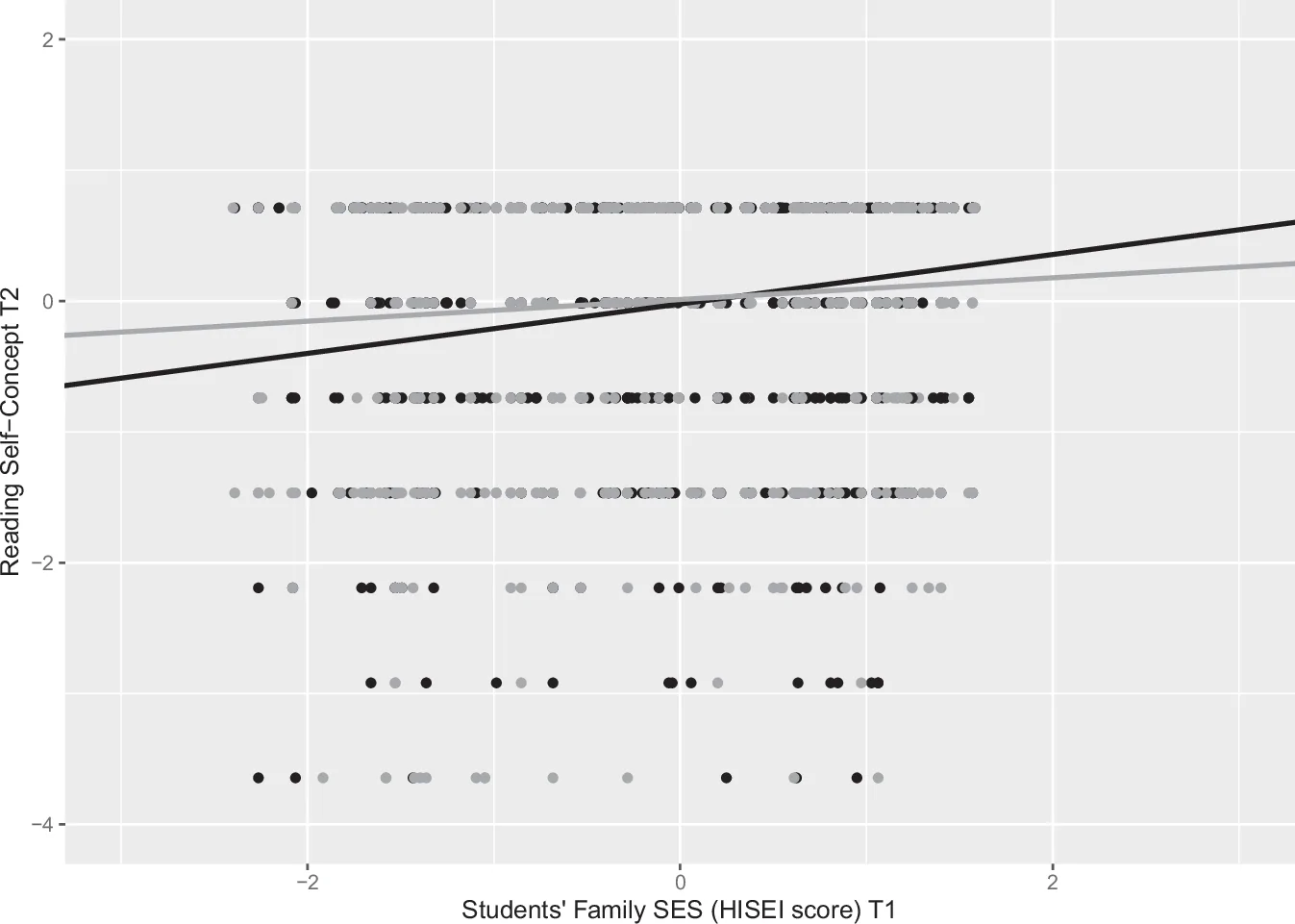
Effect of school closures on reading self-concept depending on students’ family SES. Black circles indicate observed scores in Cohort 1. Gray circles indicate observed scores in Cohort 2. The black solid line presents the expected scores based on the final estimates from the regression analysis for Cohort 1. The gray solid line presents the expected scores based on the final estimates from the regression analysis for Cohort 2. This scatterplot depicts the effect of the COVID-19-related school closures on self-concept in reading at the end of Grade 1 moderated by students’ family SES (HISEI score).

**Fig. 2:**
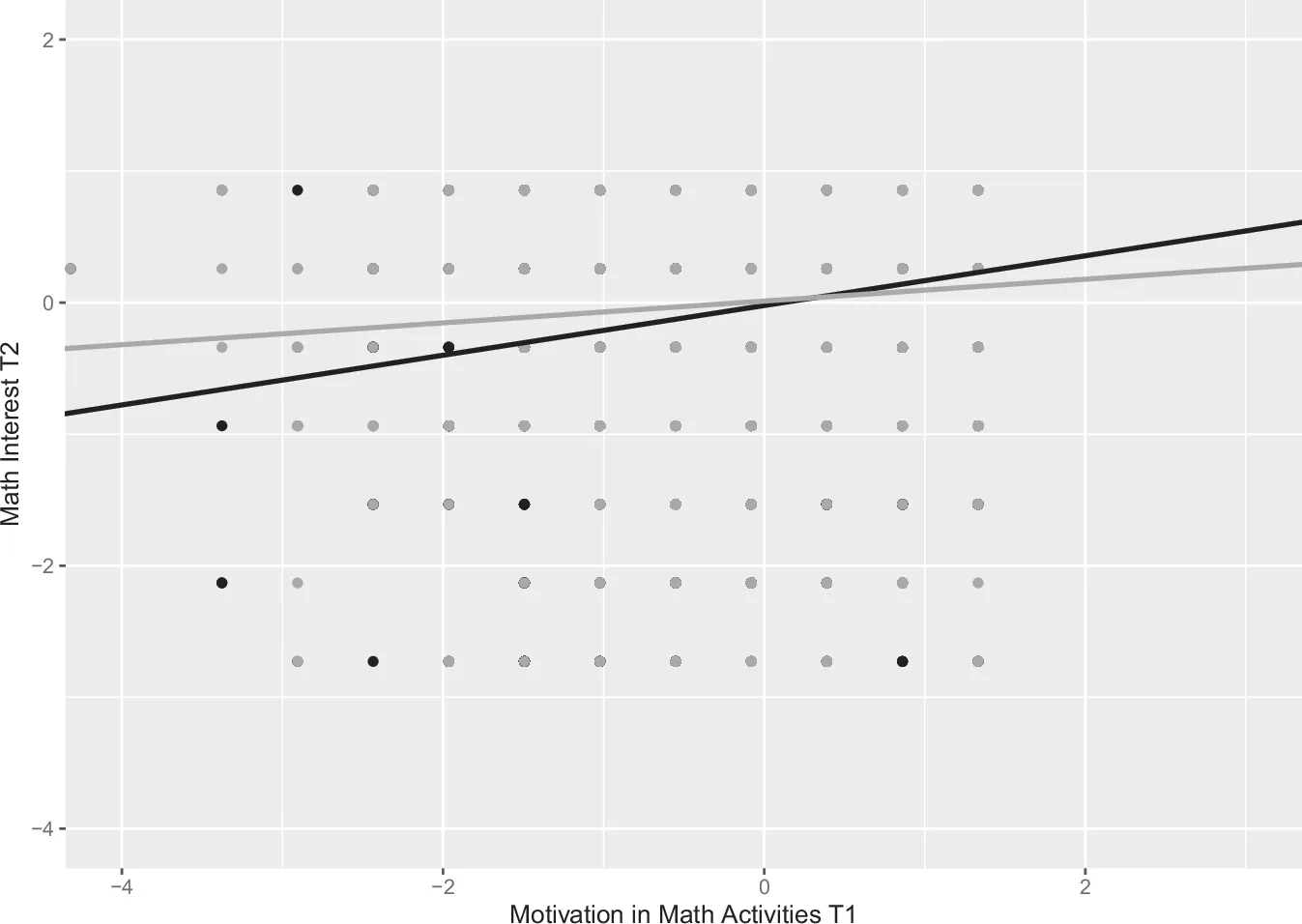
Effect of school closures on math interest depending on students’ initial motivation in math activities at the beginning of Grade 1. Black circles indicate observed scores in Cohort 1. Gray circles indicate observed scores in Cohort 2. The black solid line presents the expected scores based on the final estimates from the regression analysis for Cohort 1. The gray solid line presents the expected scores based on the final estimates from the regression analysis for Cohort 2. This scatterplot depicts the effect of the COVID-19-related school closures on interest in math at the end of Grade 1 moderated by the motivation in math activities at the beginning of Grade 1.

For reading and writing, there was a negative effect of schooling during the pandemic on the corresponding measures of intrinsic value, ${b}_{{read}}=-0.12,\,p=0.016$ and ${b}_{{write}}=-0.15,\,p=0.001$, and self-concept, ${b}_{{read}}=-0.14,p=.003$ and ${b}_{{write}}=-0.17,\,p < 0.001$, at the end of Grade 1. Thus, students in Cohort 2 evaluated their interest in reading and writing as well as their own abilities in these two domains less positively than students in Cohort 1 did when all other variables were controlled for. For math intrinsic value and self-concept, the average effects were not significantly different from zero ($p{\rm{s}}\ge 0.456$). See Table 7 for further details. Also, for the motivational variables, we tested whether students’ family SES or initial motivation moderated these effects and built corresponding interaction terms with cohort. We found that students’ family SES moderated the effect of cohort on students’ self-concept in reading, ${b}_{{read}}=0.12,{p}=.016$. Thus, for students with below-average family SES, the negative effect of the pandemic was stronger compared with their peers. In other words, the pandemic had a more deleterious effect on students’ reading self-concept for students with below-average family SES when all other baseline measures were controlled for (Fig. 1). No other interactions with students’ family SES were statistically significant (all $p{\rm{s}}\ge\left.{.071}\right)$. Furthermore, we found that students’ initial motivation in math moderated the effect of cohort on students’ interest in math at the end of Grade 1, $b_{math}=-0.11,\,p= \left.0.027\right)$. This finding indicates that students with below-average motivation to do mathematical tasks at the beginning of Grade 1 seemed to benefit from schooling during the pandemic compared with regular schooling conditions in terms of their math interest at the end of Grade 1. For further details, see Tables 8 and 9 and Fig. 2.

### Sensitivity analysis

To check for the robustness of these findings, we conducted a sensitivity analysis based on work by VanderWeele and Arah^33^. For this sensitivity analysis, we needed to determine the association between a potentially unobserved confounder and the outcome variables as well as the relationship between the unobserved confounder and the treatment variable. We assumed that the relationship between the confounder and each outcome variable could be small (*d* = 0.10), medium (*d* = 0.30), or large (*d* = 0.50). Then, we calculated the difference in the possible confounder between students in Cohort 2 and students in Cohort 1 that would be necessary to eliminate the previously observed findings. Overall, medium to large differences (*d* = 0.24 to 0.57) in an unobserved confounder between the students in Cohort 1 and students in Cohort 2 as well as a strong relationship between the confounder and the outcome variable (*d* = 0.30 or 0.50) would be necessary to eliminate the previously reported findings. For more information, see Table S1.

### The potential mechanism—effects of teacher–student contact on students’ achievement

In this analysis, we compared the effects of different levels of teacher-student contact during the pandemic with schooling before the pandemic on students’ achievement. We estimated the effects of the pandemic for three different contact conditions: low, medium, and high. For students with low teacher-student contact compared with students taught under regular schooling conditions, we expected to find negative effects of the pandemic. The estimates were negative for both reading (${b}_{{read}}=-0.21$) and mathematical achievements ${(b}_{{math}}=-0.11$), but the *p*-values were greater than 0.050 ($p{\rm{s}}\ge .163$). For more details, see Tables 10 and 11. For students with medium or high teacher-student contact compared with students taught under regular schooling conditions, we had no clear expectations. For math achievement, we surprisingly found that students with high teacher-student contact profited from learning during the pandemic compared with students under regular schooling in terms of their math achievement, ${b}_{{math}}=0.16,\,p=0.017$. This finding indicated that students who had a lot of contact with their teachers during the pandemic (i.e., more than once per week or almost every day) had higher math achievement scores at the end of Grade 1 than students who were learning under regular conditions (when all baseline measures were controlled for). The difference in the effects on math achievement between low and high teacher-student contact was also statistically significantly different from zero, ${b}_{{Ind}3-{Ind}1{math}}=0.28,{p}=.015\,$.

**Table 10:** Effects of the COVID-19 pandemic—for different levels of teacher–student contact

Variables	Indicator 1 – low contact (ES in *SD*_*Y*_) “occasionally, never”						Indicator 2 – medium contact (ES in *SD*_*Y*_) “once a week”						Indicator 3 – high contact (ES in *SD*_*Y*_) “several times per week, almost every day“					
		95% CI						95% CI						95% CI				
	b	low	up	SE	*z*	*p*	b	low	up	SE	*z*	*p*	b	low	up	SE	*z*	*p*
Achievement																		
Reading	−0.21	−0.50	0.09	0.15	−1.40	0.163	0.06	−0.10	0.22	0.08	0.70	0.481	0.14	−0.13	0.41	0.14	1.00	0.317
Mathematics	−0.11	−0.31	0.09	0.10	−1.10	0.272	−0.01	−0.11	0.10	0.06	−0.11	0.917	0.16	0.03	0.30	0.07	2.38	0.017
Motivational variables																		
Intrinsic value																		
Mathematics	0.11	−0.11	0.34	0.12	0.97	0.333	0.11	−0.03	0.26	0.07	1.54	0.123	−0.09	−0.28	0.10	0.09	−0.95	0.342
Reading	−0.22	−0.38	−0.06	0.08	−2.68	0.007	0.02	−0.12	0.17	0.07	0.31	0.754	−0.14	−0.36	0.07	0.11	−1.29	0.196
Writing	−0.18	−0.36	0.01	0.09	−1.88	0.060	−0.13	−0.29	0.02	0.08	−1.67	0.096	−0.13	−0.30	0.04	0.09	−1.51	0.132
Self−concept																		
Mathematics	0.02	−0.23	0.26	0.12	0.14	0.893	0.07	−0.08	0.22	0.08	0.94	0.348	−0.01	−0.19	0.17	0.09	−0.11	0.911
Reading	−0.30	−0.46	−0.14	0.08	−3.60	0.001	0.00	−0.15	0.15	0.08	0.04	0.967	−0.22	−0.42	−0.02	0.10	−2.13	0.033
Writing	−0.23	−0.41	−0.06	0.09	−2.62	0.009	−0.08	−0.24	0.08	0.08	−1.02	0.308	−0.19	−0.38	0.00	0.10	−1.96	0.050

Tables 10 and 11 present the findings from a multiple linear regression analysis in which the treatment condition *learning during the pandemic* was split into three subconditions: *learning during the pandemic with low teacher−student contact*, *learning during the pandemic with medium teacher−student contact*, and *learning during the pandemic with high teacher−student contact*. All dependent variables were considered in the same model. We built three indicator variables as treatment variables and used all three to predict the target outcomes. For each indicator, the reference category was regular learning (i.e., Cohort 1). Thus, Indicator 1 was created as a dummy variable to represent students in Cohort 2 who had low contact (i.e., occasional or no contact) with their teachers. The variable was coded 1 for these students and 0 for all others. Indicator 2 was coded 1 for students in Cohort 2 with medium contact with their teachers (i.e., once a week) and 0 for all others. Indicator 3 was coded 1 for students in Cohort 2 with high contact with their teachers (i.e., several times per week or almost every day) and 0 for all others. Table 10 presents the regression coefficients for the indicator variables. In this model, we controlled for any baseline differences by including all pretest measures as covariates. By using the Wald chi-square test implemented in Mplus, we could compare the effect of the school closures between the different contact conditions: low, medium, and high contact (we used the model constraint command in Mplus^62^). This is displayed in the columns labeled *Difference (ES in SD*_*Y*_*)* in Table 11. We ran the analysis in Mplus 8.5^59^. To account for potentially nonnormally distributed variables and the multilevel structure of the data (students in classes), we used cluster-robust standard errors^60^. To handle missing values, we used full information maximum likelihood (FIML) estimation. All variables were z-standardized before the analysis except for the dichotomous variables (indicator variables, gender, and migration background). Thus, the regression coefficients can be interpreted as effect sizes.

**Table 11:** Differences between the effects of the COVID-19 pandemic—for different levels of teacher–student contact

Variables	Difference Indicator 2 – Indicator 1 (ES in *SD*_*Y*_) “once a week” vs. “occasionally or never”				Difference Indicator 3 – Indicator 2 (ES in *SD*_*Y*_) “several times per week or almost every day” vs. “once a week”				Difference Indicator 3 – Indicator 1 (ES in *SD*_*Y*_) “several times per week or almost every day” vs. “occasionally or never”			
	b	SE	*z*	*p*	b	SE	*z*	*p*	b	SE	*z*	*p*
Achievement												
Reading	0.27	0.15	1.76	0.079	0.08	0.14	0.56	0.573	0.35	0.20	1.77	0.076
Mathematics	0.11	0.11	1.00	0.318	0.17	0.08	2.16	0.030	0.28	0.11	2.43	0.015
Motivational variables												
Intrinsic value												
Mathematics	0.00	0.12	0.02	0.981	−0.20	0.11	−1.87	0.062	-0.20	0.15	−1.38	0.168
Reading	0.24	0.10	2.47	0.014	−0.17	0.12	−1.36	0.172	0.07	0.13	0.57	0.569
Writing	0.05	0.12	0.38	0.703	0.00	0.11	−0.01	0.995	0.05	0.13	0.36	0.720
Self-concept												
Mathematics	0.05	0.13	0.41	0.684	−0.08	0.12	−0.68	0.496	−0.03	0.14	−0.19	0.848
Reading	0.30	0.10	3.05	0.002	−0.22	0.12	−1.79	0.073	0.08	0.12	0.64	0.522
Writing	0.15	0.11	1.40	0.163	−0.11	0.12	−0.91	0.365	0.04	0.13	0.34	0.731

Tables 10 and 11 present the findings from a multiple linear regression analysis in which the treatment condition *learning during the pandemic* was split into three subconditions: *learning during the pandemic with low teacher-student contact*, *learning during the pandemic with medium teacher-student contact*, and *learning during the pandemic with high teacher-student contact*. All dependent variables were considered in the same model. We built three indicator variables as treatment variables and used all three to predict the target outcomes. For each indicator, the reference category was regular learning (i.e., Cohort 1). Thus, Indicator 1 was created as a dummy variable to represent students in Cohort 2 who had low contact (i.e., occasional or no contact) with their teachers. The variable was coded 1 for these students and 0 for all others. Indicator 2 was coded 1 for students in Cohort 2 with medium contact with their teachers (i.e., once a week) and 0 for all others. Indicator 3 was coded 1 for students in Cohort 2 with high contact with their teachers (i.e., several times per week or almost every day) and 0 for all others. Table 10 presents the regression coefficients for the indicator variables. In this model, we controlled for any baseline differences by including all pretest measures as covariates. By using the Wald chi-square test implemented in Mplus, we could compare the effect of the school closures between the different contact conditions: low, medium, and high contact (we used the model constraint command in Mplus^62^). This is displayed in the columns labeled *Difference (ES in SD*_*Y*_*)* in Table 11. We ran the analysis in Mplus 8.5^59^. To account for potentially nonnormally distributed variables and the multilevel structure of the data (students in classes), we used cluster-robust standard errors^60^. To handle missing values, we used full information maximum likelihood (FIML) estimation. All variables were z-standardized before the analysis except for the dichotomous variables (indicator variables, gender, and migration background). Thus, the regression coefficients can be interpreted as effect sizes.

To test whether the differences between students with low, medium, or high contact with their teachers during the pandemic compared with students taught under regular conditions were moderated by students’ family SES or initial achievement, we focused on the results of the interactions between the levels of contact during the pandemic and students’ family SES or initial achievement. There was no evidence of any moderating effects of students’ family SES ($p{\rm{s}}\ge 0.137$). For more information, see Table 12. However, a difference between the coefficients of the interaction terms with baseline performance $({b}_{{Ind}2* {mathT}1} - {b}_{{Ind}1* {mathT}1})$ was statistically significant, ${b}_{{Ind}2-{Ind}1math}=-0.16,{p}=.018$. This finding is a result of a positive interaction between low teacher–student contact (Indicator 1) and baseline performance, ${b}_{{Ind}1*{mathT1}}=0.11,\,p=0.132$, and a small negative interaction between medium teacher-student contact (Indicator 2) and baseline performance, ${b}_{{Ind}2* {mathT}1}=-0.05,\,p=0.250$. This pattern indicates that, for students in the low-contact condition (compared with regular learning), a below-average baseline performance had a more negative effect on their math achievement at the end of Grade 1 than a below-average baseline performance for students with medium contact with their teachers (compared with regular learning). For more details, see Table 13.

**Table 12:** Effects of the COVID-19 pandemic on achievement—for different levels of teacher–student contact and HISEI

Variables	Mathematics (ES in *SD*_*Y*_)				Reading (ES in *SD*_*Y*_)			
	b	SE	*z*	*p*	b	SE	*z*	*p*
Predictors								
Ind 1	−0.12	0.10	−1.22	0.221	−0.22	0.14	−1.52	0.129
Ind 2	0.00	0.06	−0.02	0.984	0.07	0.08	0.85	0.393
Ind 3	0.16	0.07	2.33	0.020	0.14	0.14	1.00	0.317
HISEI	0.05	0.03	2.01	0.044	0.11	0.03	3.27	0.001
Ind 1*HISEI	-0.04	0.07	−0.62	0.538	-	-	-	-
Ind 2*HISEI	-0.03	0.06	−0.39	0.696	-	-	-	-
Ind 3*HISEI	0.09	0.06	1.49	0.137	-	-	-	-
Difference Variables								
Ind 2 – Ind 1	0.12	0.10	1.15	0.249	0.29	0.15	1.96	0.050
Ind 3 – Ind 2	0.17	0.08	2.07	0.038	0.07	0.15	0.47	0.642
Ind 3 – Ind 1	0.28	0.11	2.53	0.011	0.35	0.19	1.87	0.062
Ind 2*HISEI – Ind1*HISEI	0.02	0.09	0.18	0.855	-	-	-	-
Ind 3*HISEI – Ind2*HISEI	0.11	0.08	1.47	0.142	-	-	-	-
Ind 3*HISEI – Ind1*HISEI	0.13	0.08	1.56	0.119	-	-	-	-

Ind 1: Indicator 1 – low contact. Ind 2: Indicator 2 – medium contact. Ind 3: Indicator 3 – high contact. The table presents the findings from a multiple linear regression analysis in which the treatment condition *learning during the pandemic* was split into three subconditions: *learning during the pandemic with low teacher-student contact*, *learning during the pandemic with medium teacher-student contact*, and *learning during the pandemic with high teacher-student contact*. All dependent variables were considered in the same model. We built three indicator variables as treatment variables and used all three to predict the target outcomes. For each indicator, the reference category was regular learning (i.e., Cohort 1). Thus, Indicator 1 was created as a dummy variable to represent students in Cohort 2 who had low contact (i.e., occasional or no contact) with their teachers. The variable was coded 1 for these students and 0 for all others. Indicator 2 was coded 1 for students in Cohort 2 with medium contact with their teachers (i.e., once a week) and 0 for all others. Indicator 3 was coded 1 for students in Cohort 2 with high contact with their teachers (i.e., several times per week or almost every day) and 0 for all others. The regression coefficients for the indicator variables, the HISEI score, and the respective interaction terms are shown in this table. In this model, we controlled for any baseline differences by including all pretest measures as covariates. By using the Wald chi-square test implemented in Mplus, we could compare the effects of the school closures for students with low, medium, and high contact with their teachers as well as the interaction terms (we used the model constraint command in Mplus^62^). These results are presented in the rows labeled *Difference Variables*. We ran the analysis in Mplus 8.5^59^. To account for potentially nonnormally distributed variables and the multilevel structure of the data (students nested in classes), we used cluster-robust standard errors^60^. To handle missing values, we used full information maximum likelihood (FIML) estimation. All variables were z-standardized before the analysis except for the dichotomous variables (indicator variables, gender, and migration background). Thus, the regression coefficients can be interpreted as effect sizes.

**Table 13:** Effects of the COVID-19 pandemic on achievement—for different levels of teacher–student contact and the baseline score

Variables	Mathematics (ES in *SD*_*Y*_)				Reading (ES in *SD*_*Y*_)			
	b	SE	*z*	*p*	b	SE	*z*	*p*
Predictors								
Ind 1	−0.11	0.10	−1.11	0.268	−0.22	0.15	−1.46	0.143
Ind 2	−0.01	0.05	−0.24	0.812	0.05	0.08	0.66	0.510
Ind 3	0.16	0.08	2.12	0.034	0.17	0.14	1.27	0.203
Baseline	0.58	0.03	20.36	<0.001	0.09	0.02	5.51	<0.001
Ind 1* Baseline	0.11	0.07	1.51	0.132	-	-	-	-
Ind 2* Baseline	−0.05	0.05	−1.15	0.250	-	-	-	-
Ind 3* Baseline	−0.01	0.09	−0.16	0.876				
Difference Variables								
Ind 2 – Ind 1	0.10	0.11	0.96	0.340	0.27	0.15	1.79	0.073
Ind 3 – Ind 2	0.18	0.09	2.07	0.039	0.12	0.14	0.85	0.397
Ind 3 – Ind 1	0.28	0.12	2.33	0.020	0.39	0.19	2.01	0.044
Ind 2* Baseline – Ind 1* Baseline	−0.16	0.07	-2.37	0.018	-	-	-	-
Ind 3* Baseline – Ind 2* Baseline	0.04	0.10	0.42	0.678				
Ind 3* Baseline – Ind 1* Baseline	−0.12	0.10	−1.16	0.247				

Ind 1: Indicator 1 – low contact. Ind 2: Indicator 2 – medium contact. Ind 3: Indicator 3 – high contact. The table presents the findings from a multiple linear regression analysis in which the treatment condition *learning during the pandemic* was split into three subconditions: *learning during the pandemic with low teacher-student contact*, *learning during the pandemic with medium teacher-student contact*, and *learning during the pandemic with high teacher-student contact*. All dependent variables were considered in the same model. We built three indicator variables as treatment variables and used all three to predict the target outcomes. For each indicator, the reference category was regular learning (i.e., Cohort 1). Thus, Indicator 1 was created as a dummy variable to represent students in Cohort 2 who had low contact (i.e., occasional or no contact) with their teachers. The variable was coded 1 for these students and 0 for all others. Indicator 2 was coded 1 for students in Cohort 2 with medium contact with their teachers (i.e., once a week) and 0 for all others. Indicator 3 was coded 1 for students in Cohort 2 with high contact with their teachers (i.e., several times per week or almost every day) and 0 for all others. The regression coefficients for the indicator variables, the pretest score, and the respective interaction terms are shown in this table. In this model, we controlled for any baseline differences by including all pretest measures as covariates. By using the Wald chi-square test implemented in Mplus, we could compare the effect of the school closures for students with low, medium, and high contact with their teachers as well as the interaction terms (we used the model constraint command in Mplus^62^). These results are presented in the rows labeled *Difference Variables*. We ran the analysis in Mplus 8.5^59^. To account for potentially nonnormally distributed variables and the multilevel structure of the data (students nested in classes), we used cluster-robust standard errors^60^. To handle missing values, we used full information maximum likelihood (FIML) estimation. All variables were z-standardized before the analysis except for the dichotomous variables (indicator variables, gender, and migration background). Thus, the regression coefficients can be interpreted as effect sizes.

### The potential mechanism—effects of teacher–student contact on students’ motivation

We compared the effects of different levels of teacher-student contact during the pandemic with schooling before the pandemic on the motivational variables, too. Similar to the average effects of the pandemic on students’ motivation, we found negative effects on students’ intrinsic value and self-concept for reading and writing in the low as well as high contact conditions (compared with regular learning). In the low-contact condition, we observed negative effects on students’ intrinsic value in reading, ${b}_{{read}}=-0.22,\,p=0.007$, and writing, ${b}_{{write}}=-0.18,\,p=0.060$, and on students’ self-concepts in reading, ${b}_{{read}}=-0.30,\,p=0.001$, and writing, ${b}_{{write}}=-0.23,{p}=0.009$. Furthermore, students who had high teacher-student contact during the pandemic compared with students taught under regular learning conditions also reported lower self-concept scores in reading and writing at the end of Grade 1, ${b}_{{read}}=-0.22,p=0.033$ and ${b}_{{write}}=-0.19,\,{p}=0.050$. These negative effects on motivation did not differ substantially between the low and high teacher-student contact conditions. For the medium contact condition, the regression coefficients were close to zero and were not statistically significant. For more details, see Tables 10 and 11.

**Fig. 3:**
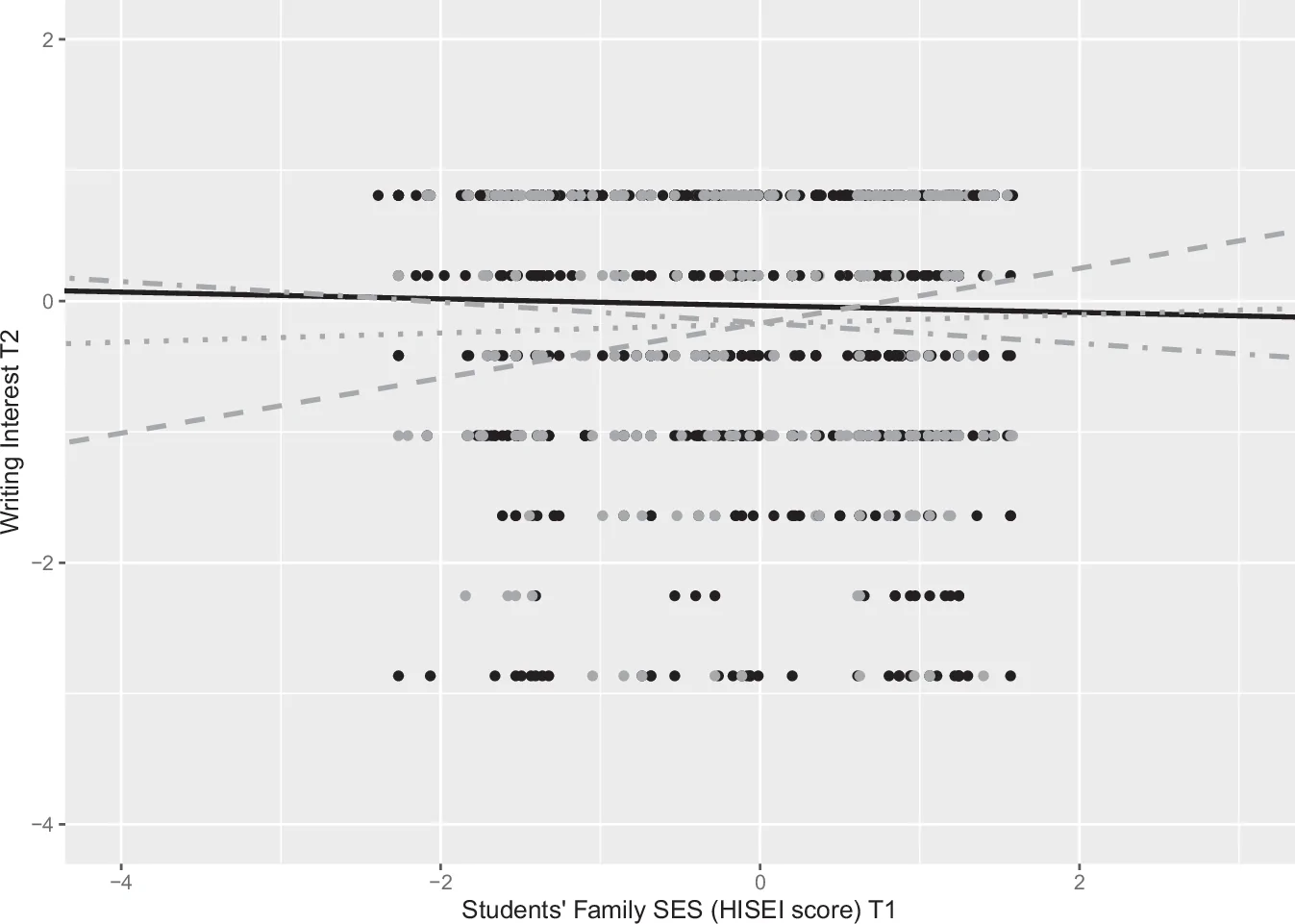
Effects of the levels of teacher–student contact during school closures on interest in writing depending on students’ family SES. Black circles indicate observed scores in Cohort 1. Gray circles indicate observed scores in Cohort 2. The black solid line presents the expected scores based on the final estimates from the regression analysis for Cohort 1. The gray dashed line presents the expected scores based on the final estimates from the regression analysis for Cohort 2 with low teacher-student contact. The gray dotted line presents the expected scores based on the final estimates from the regression analysis for Cohort 2 with medium teacher-student contact. The gray dot-dashed line presents the expected scores based on the final estimates from the regression analysis for Cohort 2 with high teacher–student contact. This scatterplot depicts the effects of the levels of teacher-student contact during the COVID-19-related school closures on interest in writing at the end of Grade 1 moderated by students’ family SES (HISEI score).

**Fig. 4:**
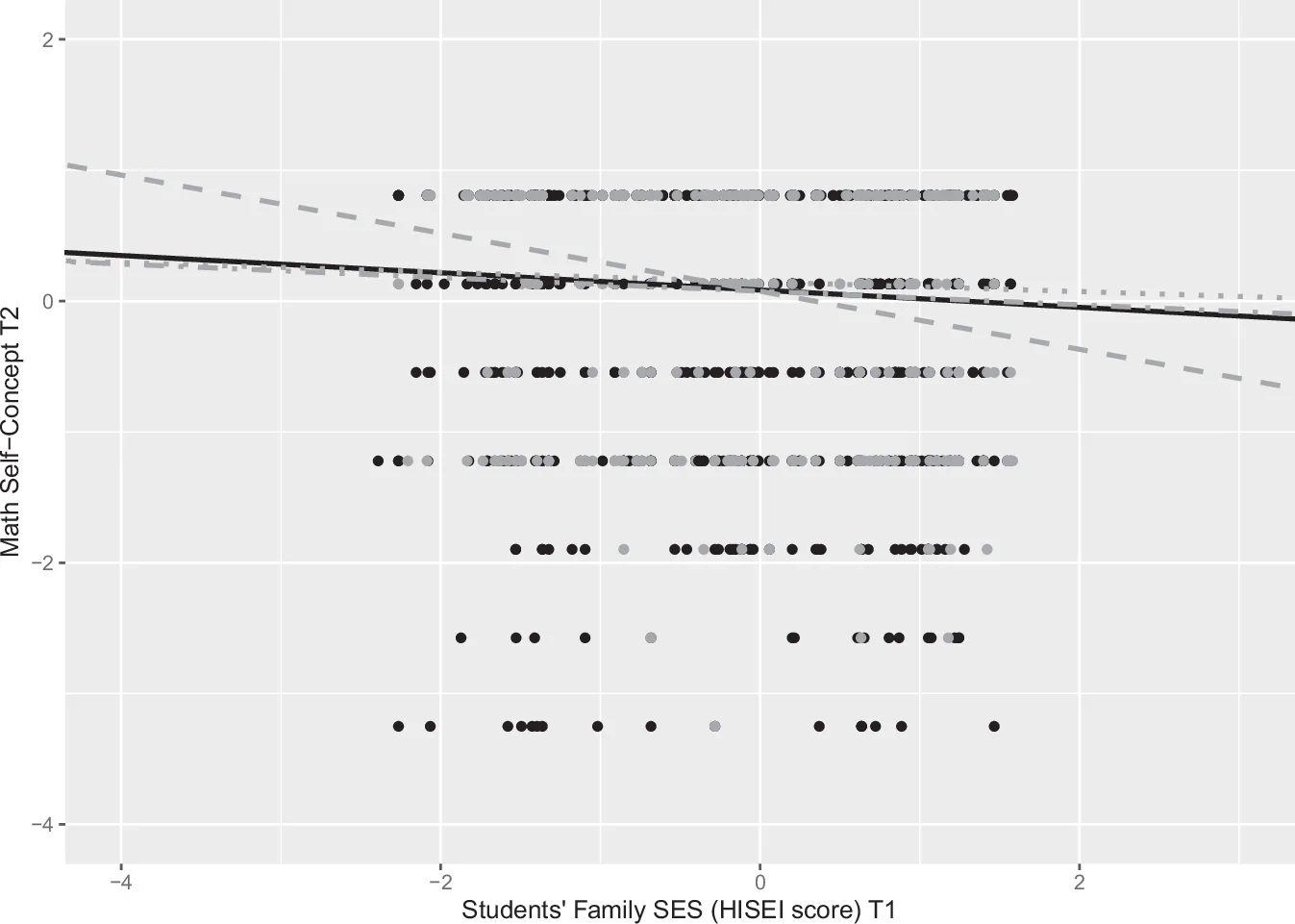
Effects of the levels of teacher–student contact during school closures on in math self-concept depending on students’ family SES. Black circles indicate observed scores in Cohort 1. Gray circles indicate observed scores in Cohort 2. The black solid line presents the expected scores based on the final estimates from the regression analysis for Cohort 1. The gray dashed line presents the expected scores based on the final estimates from the regression analysis for Cohort 2 with low teacher-student contact. The gray dotted line presents the expected scores based on the final estimates from the regression analysis for Cohort 2 with medium teacher-student contact. The gray dot-dashed line presents the expected scores based on the final estimates from the regression analysis for Cohort 2 with high teacher-student contact. This scatterplot depicts the effects of the levels of teacher-student contact during the COVID-19-related school closures on self-concept in math at the end of Grade 1 moderated by students’ family SES (HISEI score).

To investigate whether students’ family SES or initial motivation moderated these findings, the interactions between the indicator variables and family SES or initial motivation were considered. We found that students with low contact with their teachers (i.e., occasionally or never) and who came from families with below-average SES suffered more disadvantages during the pandemic in terms of intrinsic value in writing compared with students from high SES families, ${b}_{{write}}=0.24,{p}=0.024$ (see Fig. 3). For self-concept in math, the pattern was reversed, ${b}_{{math}}=-0.16,\,p=0.041$. Above-average family SES was associated with a disadvantage for students’ self-concept in math for students in the low-contact condition compared with students’ learning under regular conditions (see Fig. 4). A comparison of interaction terms for students with low versus high teacher-student contact compared with regular learning conditions strengthened the finding and revealed significant differences for interest in writing, ${b}_{{Ind}3-{Ind}1{write}}=-0.29,\,p=0.045$ and self-concept in math, ${b}_{{Ind}2-{Ind}1{math}}=0.18,{p}=.050$. For more details, see Tables 14 and 15.

**Table 14:** Effects of the COVID-19 pandemic on intrinsic value—for different levels of teacher-student contact and HISEI

Variables	Mathematics (ES in *SD*_*Y*_)				Reading (ES in *SD*_*Y*_)				Writing (ES in *SD*_*Y*_)			
	b	SE	*z*	*p*	b	SE	*z*	*p*	b	SE	*z*	*p*
Predictors												
Ind 1	0.11	0.12	0.92	0.356	−0.20	0.09	−2.37	0.018	−0.13	0.10	−1.36	0.174
Ind 2	0.11	0.08	1.39	0.166	0.01	0.08	0.10	0.919	−0.14	0.08	−1.70	0.089
Ind 3	−0.09	0.10	−0.94	0.345	−0.14	0.11	−1.29	0.199	−0.13	0.09	−1.47	0.143
HISEI	−0.10	0.03	−3.04	0.002	0.07	0.04	1.93	0.054	−0.03	0.04	−0.68	0.499
Ind 1*HISEI	−0.03	0.08	−0.42	0.677	0.11	0.11	1.04	0.298	0.24	0.10	2.25	0.024
Ind 2*HISEI	0.03	0.07	0.42	0.675	0.12	0.10	1.25	0.212	0.06	0.09	0.68	0.498
Ind 3*HISEI	0.00	0.10	0.01	0.995	0.03	0.14	0.24	0.812	−0.05	0.11	−0.48	0.629
Difference Variables												
Ind 2 – Ind 1	0.00	0.13	−0.02	0.983	0.21	0.11	2.01	0.044	0.00	0.12	−0.03	0.976
Ind 3 – Ind 2	−0.20	0.11	−1.72	0.085	−0.15	0.12	−1.24	0.217	0.01	0.11	0.05	0.961
Ind 3 – Ind 1	−0.20	0.15	−1.31	0.189	0.06	0.13	0.46	0.644	0.00	0.13	0.01	0.989
Ind 2*HISEI – Ind1*HISEI	0.07	0.11	0.62	0.536	0.01	0.15	0.10	0.921	−0.18	0.14	−1.26	0.208
Ind 3*HISEI – Ind2*HISEI	−0.03	0.11	−0.29	0.771	−0.09	0.16	−0.57	0.566	−0.11	0.14	−0.83	0.406
Ind 3*HISEI – Ind1*HISEI	0.04	0.12	0.29	0.771	−0.08	0.18	−0.43	0.668	−0.29	0.15	−2.00	0.045

Ind 1: Indicator 1 – low contact. Ind 2: Indicator 2 – medium contact. Ind 3: Indicator 3 – high contact. The table presents the findings from a multiple linear regression analysis in which the treatment condition *learning during the pandemic* was split into three subconditions: *learning during the pandemic with low teacher−student contact*, *learning during the pandemic with medium teacher−student contact*, and *learning during the pandemic with high teacher−student contact*. All dependent variables were considered in the same model. We built three indicator variables as treatment variables and used all three to predict the target outcomes. For each indicator, the reference category was regular learning (i.e., Cohort 1). Thus, Indicator 1 was created as a dummy variable to represent students in Cohort 2 who had low contact (i.e., occasional or no contact) with their teachers. The variable was coded 1 for these students and 0 for all others. Indicator 2 was coded 1 for students in Cohort 2 with medium contact with their teachers (i.e., once a week) and 0 for all others. Indicator 3 was coded 1 for students in Cohort 2 with high contact with their teachers (i.e., several times per week or almost every day) and 0 for all others. The regression coefficients for the indicator variables, the HISEI score, and the respective interaction terms are shown in this table. In this model, we controlled for any baseline differences by including all pretest measures as covariates. By using the Wald chi-square test implemented in Mplus, we could compare the effects of the school closures for students with low, medium, and high contact with their teachers as well as the interaction terms (we used the model constraint command in Mplus^62^). These results are presented in the rows labeled *Difference Variables*. We ran the analysis in Mplus 8.5^59^. To account for potentially nonnormally distributed variables and the multilevel structure of the data (students nested in classes), we used cluster-robust standard errors^60^. To handle missing values, we used full information maximum likelihood (FIML) estimation. All variables were z-standardized before the analysis except for the dichotomous variables (indicator variables, gender, and migration background). Thus, the regression coefficients can be interpreted as effect sizes.

**Table 15:** Effects of the COVID-19 pandemic on self-concept—for different levels of teacher–student contact and HISEI

Variables	Mathematics (ES in *SD*_*Y*_)				Reading (ES in *SD*_*Y*_)				Writing (ES in *SD*_*Y*_)			
	b	SE	*z*	*p*	b	SE	*z*	*p*	b	SE	*z*	*p*
Predictors												
Ind 1	−0.01	0.13	−0.07	0.944	−0.29	0.09	−3.25	0.001	−0.20	0.10	−2.01	0.045
Ind 2	0.06	0.08	0.78	0.435	−0.01	0.08	−0.11	0.917	−0.07	0.08	−0.83	0.408
Ind 3	−0.01	0.09	−0.10	0.921	−0.22	0.10	−2.13	0.033	−0.19	0.10	−1.87	0.062
HISEI	−0.07	0.04	−1.78	0.075	0.08	0.03	2.53	0.011	0.06	0.03	1.86	0.063
Ind 1*HISEI	−0.16	0.08	−2.05	0.041	0.09	0.10	0.89	0.374	0.14	0.12	1.24	0.215
Ind 2*HISEI	0.03	0.07	0.40	0.689	0.10	0.11	0.91	0.365	−0.09	0.11	−0.85	0.397
Ind 3*HISEI	0.01	0.11	0.13	0.897	0.07	0.15	0.46	0.648	−0.17	0.10	−1.73	0.084
Difference Variables												
Ind 2 – Ind 1	0.07	0.14	0.51	0.607	0.28	0.11	2.55	0.011	0.14	0.12	1.15	0.252
Ind 3 – Ind 2	−0.07	0.12	−0.57	0.566	−0.21	0.13	−1.66	0.098	−0.12	0.13	−0.97	0.334
Ind 3 – Ind 1	0.00	0.15	0.00	0.998	0.07	0.13	0.54	0.590	0.02	0.14	0.11	0.912
Ind 2*HISEI – Ind1*HISEI	0.18	0.09	1.96	0.050	0.01	0.15	0.06	0.956	−0.23	0.14	−1.63	0.104
Ind 3*HISEI – Ind2*HISEI	−0.02	0.12	−0.13	0.897	−0.03	0.19	−0.18	0.861	−0.08	0.14	−0.57	0.571
Ind 3*HISEI – Ind1*HISEI	0.17	0.12	1.40	0.161	−0.03	0.17	−0.15	0.884	−0.31	0.14	−2.21	0.027

Ind 1: Indicator 1 – low contact. Ind 2: Indicator 2 – medium contact. Ind 3: Indicator 3 – high contact. The table presents the findings from a multiple linear regression analysis in which the treatment condition *learning during the pandemic* was split into three subconditions: *learning during the pandemic with low teacher−student contact*, *learning during the pandemic with medium teacher−student contact*, and *learning during the pandemic with high teacher−student contact*. All dependent variables were considered in the same model. We built three indicator variables as treatment variables and used all three to predict the target outcomes. For each indicator, the reference category was regular learning (i.e., Cohort 1). Thus, Indicator 1 was created as a dummy variable to represent students in Cohort 2 who had low contact (i.e., occasional or no contact) with their teachers. The variable was coded 1 for these students and 0 for all others. Indicator 2 was coded 1 for students in Cohort 2 with medium contact with their teachers (i.e., once a week) and 0 for all others. Indicator 3 was coded 1 for students in Cohort 2 with high contact with their teachers (i.e., several times per week or almost every day) and 0 for all others. The regression coefficients for the indicator variables, the HISEI score, and the respective interaction terms are shown in this table. In this model, we controlled for any baseline differences by including all pretest measures as covariates. By using the Wald chi-square test implemented in Mplus, we could compare the effects of the school closures for students with low, medium, and high contact with their teachers as well as the interaction terms (we used the model constraint command in Mplus^62^). These results are presented in the rows labeled *Difference Variables*. We ran the analysis in Mplus 8.5^59^. To account for potentially nonnormally distributed variables and the multilevel structure of the data (students nested in classes), we used cluster-robust standard errors^60^. To handle missing values, we used full information maximum likelihood (FIML) estimation. All variables were z-standardized before the analysis except for the dichotomous variables (indicator variables, gender, and migration background). Thus, the regression coefficients can be interpreted as effect sizes.

**Fig. 5:**
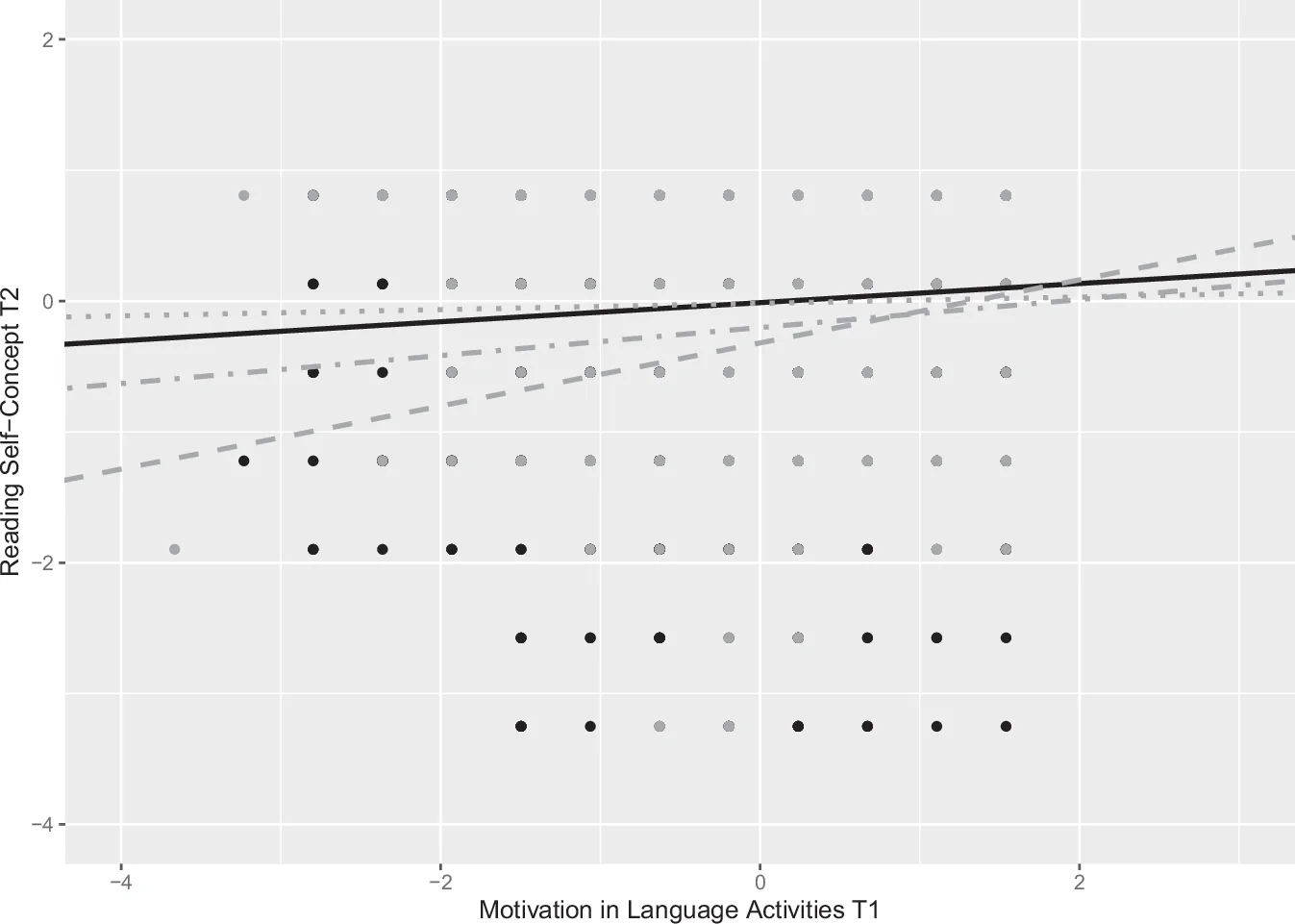
Effects of the levels of teacher–student contact during school closures on reading self-concept depending on students’ initial motivation in language activities at the beginning of Grade 1. Black circles indicate observed scores in Cohort 1. Gray circles indicate observed scores in Cohort 2. The black solid line presents the expected scores based on the final estimates from the regression analysis for Cohort 1. The gray dashed line presents the expected scores based on the final estimates from the regression analysis for Cohort 2 with low teacher-student contact. The gray dotted line presents the expected scores based on the final estimates from the regression analysis for Cohort 2 with medium teacher-student contact. The gray dot-dashed line presents the expected scores based on the final estimates from the regression analysis for Cohort 2 with high teacher-student contact. This scatterplot depicts the effects of the levels of teacher-student contact during the COVID-19-related school closures on self-concept in reading at the end of Grade 1 moderated by motivation in language activities at the beginning of Grade 1.

For initial motivation, we found that the interaction between low teacher-student contact and students’ initial motivation was statistically significant for self-concept in reading, ${b}_{{read}}=0.17,{p}=.009$. This finding indicates that below-average baseline motivation seemed to be a risk factor during the pandemic in the condition with low teacher-student contact for students’ self-concept in reading (see Fig. 5). A comparison of interaction terms for students with low versus medium contact compared with regular learning conditions strengthened the finding and revealed a significant difference for self-concept in reading, ${b}_{{Ind}2-{Ind}1{read}}=-0.22,\,p=0.014$. No other interactions were statistically significant no matter whether students had low or high contact with their teachers (all $p{\rm{s}}\ge 0.185$). For more details, see Tables 16 and 17.

**Table 16:** Effects of the COVID-19 pandemic on intrinsic value—for different levels of teacher-student contact and baseline score

Variables	Mathematics (ES in *SD*_*Y*_)				Reading (ES in *SD*_*Y*_)				Writing (ES in *SD*_*Y*_)			
	b	SE	*z*	*p*	b	SE	*z*	*p*	b	SE	*z*	*p*
Predictors												
Ind 1	0.12	0.12	0.97	0.331	−0.22	0.08	−2.73	0.006	−0.19	0.09	−1.97	0.049
Ind 2	0.11	0.08	1.47	0.141	0.01	0.08	0.18	0.856	−0.13	0.08	−1.67	0.095
Ind 3	−0.11	0.10	−1.07	0.283	−0.12	0.12	−1.03	0.304	−0.12	0.09	−1.28	0.202
Baseline	0.19	0.04	4.35	<0.001	0.06	0.04	1.62	0.106	0.08	0.03	2.25	0.025
Ind 1* Baseline	−0.06	0.11	−0.54	0.591	0.05	0.10	0.51	0.614	0.04	0.07	0.57	0.572
Ind 2* Baseline	−0.09	0.06	−1.47	0.142	−0.01	0.08	−0.14	0.887	0.07	0.09	0.78	0.433
Ind 3* Baseline	−0.11	0.09	−1.24	0.216	0.11	0.10	1.15	0.249	−0.04	0.07	−0.62	0.537
Difference Variables												
Ind 2 – Ind 1	−0.01	0.12	−0.04	0.969	0.23	0.10	2.36	0.018	0.06	0.12	0.48	0.635
Ind 3 – Ind 2	−0.22	0.11	−1.88	0.060	−0.14	0.13	−1.03	0.302	0.01	0.11	0.10	0.918
Ind 3 – Ind 1	−0.22	0.15	−1.47	0.141	0.10	0.13	0.73	0.466	0.07	0.13	0.54	0.592
Ind 2* Baseline –												
Ind 1* Baseline	−0.03	0.11	−0.27	0.789	−0.06	0.11	−0.52	0.605	0.03	0.09	0.34	0.735
Ind 3* Baseline –												
Ind 2* Baseline	−0.03	0.10	−0.28	0.783	0.12	0.13	0.96	0.339	−0.11	0.12	−0.98	0.329
Ind 3* Baseline –												
Ind 1* Baseline	−0.06	0.13	−0.42	0.674	0.06	0.12	0.51	0.613	−0.08	0.10	−0.85	0.395

Ind 1: Indicator 1 – low contact. Ind 2: Indicator 2 – medium contact. Ind 3: Indicator 3 – high contact. The table presents the findings from a multiple linear regression analysis in which the treatment condition *learning during the pandemic* was split into three subconditions: *learning during the pandemic with low teacher−student contact*, *learning during the pandemic with medium teacher−student contact*, and *learning during the pandemic with high teacher−student contact*. All dependent variables were considered in the same model. We built three indicator variables as treatment variables and used all three to predict the target outcomes. For each indicator, the reference category was regular learning (i.e., Cohort 1). Thus, Indicator 1 was created as a dummy variable to represent students in Cohort 2 who had low contact (i.e., occasional or no contact) with their teachers. The variable was coded 1 for these students and 0 for all others. Indicator 2 was coded 1 for students in Cohort 2 with medium contact with their teachers (i.e., once a week) and 0 for all others. Indicator 3 was coded 1 for students in Cohort 2 with high contact with their teachers (i.e., several times per week or almost every day) and 0 for all others. The regression coefficients for the indicator variables, the pretest score, and the respective interaction terms are shown in this table. In this model, we controlled for any baseline differences by including all pretest measures as covariates. By using the Wald chi-square test implemented in Mplus, we could compare the effect of the school closures for students with low, medium, and high contact with their teachers as well as the interaction terms (we used the model constraint command in Mplus^62^). These results are presented in the rows labeled *Difference Variables*. We ran the analysis in Mplus 8.5^59^. To account for potentially nonnormally distributed variables and the multilevel structure of the data (students nested in classes), we used cluster-robust standard errors^60^. To handle missing values, we used full information maximum likelihood (FIML) estimation. All variables were z-standardized before the analysis except for the dichotomous variables (indicator variables, gender, and migration background). Thus, the regression coefficients can be interpreted as effect sizes.

**Table 17:** Effects of the COVID-19 pandemic on self-concept—for different levels of teacher–student contact and baseline score

Variables	Mathematics (ES in *SD*_*Y*_)				Reading (ES in *SD*_*Y*_)				Writing (ES in *SD*_*Y*_)			
	b	SE	*z*	*p*	b	SE	*z*	*p*	b	SE	*z*	*p*
Predictors												
Ind 1	0.02	0.12	0.15	0.880	−0.31	0.08	−3.73	<0.001	−0.25	0.10	−2.58	0.010
Ind 2	0.07	0.08	0.84	0.402	−0.01	0.08	−0.07	0.947	−0.07	0.08	−0.88	0.382
Ind 3	−0.04	0.11	−0.34	0.731	−0.19	0.11	−1.74	0.083	−0.18	0.10	−1.69	0.091
Baseline	0.15	0.04	3.40	0.001	0.07	0.03	2.32	0.020	0.06	0.03	1.94	0.052
Ind 1* Baseline	−0.05	0.11	−0.45	0.656	0.17	0.06	2.63	0.009	0.08	0.09	0.93	0.350
Ind 2* Baseline	−0.10	0.06	−1.56	0.119	−0.05	0.08	−0.65	0.518	−0.02	0.10	−0.24	0.813
Ind 3* Baseline	−0.09	0.11	−0.87	0.385	0.03	0.08	0.43	0.668	−0.02	0.08	−0.27	0.786
Difference Variables												
Ind 2 – Ind 1	0.05	0.13	0.35	0.729	0.30	0.10	3.05	0.002	0.17	0.12	1.49	0.135
Ind 3 – Ind 2	−0.10	0.13	−0.78	0.437	−0.19	0.13	−1.41	0.157	−0.11	0.13	−0.81	0.416
Ind 3 – Ind 1	−0.06	0.15	−0.37	0.712	0.12	0.13	0.91	0.361	0.07	0.14	0.51	0.610
Ind 2* Baseline – Ind 1* Baseline	−0.05	0.12	−0.44	0.659	−0.22	0.09	−2.46	0.014	−0.11	0.11	−0.94	0.348
Ind 3* Baseline – Ind 2* Baseline	0.01	0.11	0.06	0.950	0.08	0.11	0.75	0.456	0.00	0.12	0.01	0.992
Ind 3* Baseline – Ind 1* Baseline	−0.05	0.14	−0.32	0.751	−0.13	0.10	−1.38	0.167	−0.10	0.10	−1.01	0.314

Ind 1: Indicator 1 – low contact. Ind 2: Indicator 2 – medium contact. Ind 3: Indicator 3 – high contact. The table presents the findings from a multiple linear regression analysis in which the treatment condition *learning during the pandemic* was split into three subconditions: *learning during the pandemic with low teacher-student contact*, *learning during the pandemic with medium teacher-student contact*, and *learning during the pandemic with high teacher-student contact*. All dependent variables were considered in the same model. We built three indicator variables as treatment variables and used all three to predict the target outcomes. For each indicator, the reference category was regular learning (i.e., Cohort 1). Thus, Indicator 1 was created as a dummy variable to represent students in Cohort 2 who had low contact (i.e., occasional or no contact) with their teachers. The variable was coded 1 for these students and 0 for all others. Indicator 2 was coded 1 for students in Cohort 2 with medium contact with their teachers (i.e., once a week) and 0 for all others. Indicator 3 was coded 1 for students in Cohort 2 with high contact with their teachers (i.e., several times per week or almost every day) and 0 for all others. The regression coefficients for the indicator variables, the pretest score, and the respective interaction terms are shown in this table. In this model, we controlled for any baseline differences by including all pretest measures as covariates. By using the Wald chi-square test implemented in Mplus, we could compare the effect of the school closures for students with low, medium, and high contact with their teachers as well as the interaction terms (we used the model constraint command in Mplus^62^). These results are presented in the rows labeled *Difference Variables*. We ran the analysis in Mplus 8.5^59^. To account for potentially nonnormally distributed variables and the multilevel structure of the data (students nested in classes), we used cluster-robust standard errors^60^. To handle missing values, we used full information maximum likelihood (FIML) estimation. All variables were z-standardized before the analysis except for the dichotomous variables (indicator variables, gender, and migration background). Thus, the regression coefficients can be interpreted as effect sizes.

### Robustness checks

In all analyses with different subsets, the overall pattern of results was generally comparable to the results reported in the main text. The findings in the additional analyses revealed even stronger findings that were more in line with our expectations to some extent. Only the analyses of the data set containing the 19 schools that had participants in both cohorts revealed a slightly different picture: The regression coefficients of the differences between the two cohorts were close to zero. However, the pattern of findings was comparable if low teacher-student contact was considered. We do not overemphasize this difference because the sample size was small. See Tables S2–S16 for further information.

## Discussion

In this study, we were particularly interested in whether teaching-specific factors would be found to mitigate the expected, negative consequences of schooling during the first wave of the pandemic on students’ achievement and motivation at the beginning of compulsory schooling. We investigated the role of teacher–student contact as one of the most psychologically relevant variables during that time. To summarize the effects on school achievement, we did not find average negative effects of learning under pandemic conditions compared with learning under regular conditions on students’ school achievement at the end of Grade 1. The 95% confidence intervals implied that small negative to small positive effects were compatible with the data for reading [−0.11, 0.16] and math [−0.10, 0.08]. Furthermore, we did not observe that students’ family SES or initial achievement moderated these effects. Interestingly, there was a small positive effect of high contact between teachers and students during the school closures (vs. regular schooling) on math achievement, 95% CI [0.03, 0.30]. This positive effect on math achievement—when students had a high level of contact with their teachers—might indicate a substantial compensation effect when teachers were able not only to provide learning materials but also to interact with students, parents, or both; to provide feedback; and to be responsive to their students^34^.

For motivational outcomes, there were substantial average negative effects of the pandemic-related school closures on students’ interest and self-concepts in reading and writing. And the negative effect on students’ self-concept in reading was even larger for students from low-SES families compared with those from high-SES families and students who had little contact with their teachers. Students were probably missing the support, reinforcement, and feedback they would have experienced in class compared with their learning environment at home, especially if their family SES and contact with teachers were low. These findings are in line with expectations drawn from the findings of single studies that used parent reports or self-reports from older students^9,13,35,36^. Overall, the negative effects on students’ motivation might be explained by a reduction or impairment in teaching quality and teacher support due to the lockdown and the changes in the teaching situation in the subsequent weeks^25,26^. Low and high teacher-student contact (as one proxy for teacher support) during the pandemic were linked to negative effects on students’ motivation. According to expectancy-value theory^16^, teachers are socializers who use feedback to impact students’ perceptions of their own abilities^37–39^ and thus affect students’ task values (e.g., intrinsic value) and expectations for success (i.e., self-concept). The negative effects on students’ motivation are concerning, and they might be associated with negative effects on achievement and achievement-related choices later on^40–44^.

This study is unique in the strengths of its research design, measurement occasions, and sample. Nevertheless, there are methodological issues that need to be considered when interpreting the results. First, the sample was a convenience sample that was not randomly drawn from the population of first graders in Baden-Württemberg. The characteristics of our sample—compared with official statistics—indicate a slightly positive selection effect in terms of class size, migration background, language spoken at home, and family SES. This composition might contribute to explaining the absence of average negative effects of the pandemic on students’ achievement because schools and families had more resources—compared with the national and international averages—to face the challenges of the pandemic. Nevertheless, our data are unique, as studies comparing first graders before and during the pandemic are very rare. Second, there was a technical problem in Cohort 1. The most difficult reading tasks were not presented to some of the children due to a specific response pattern. Thus, the findings related to reading achievement need to be interpreted with caution, although the procedure chosen to handle the technical problem seems to be appropriate for the beginning and end of Grade 1. Third, the assessment situation at the end of Grade 1 was not fully identical between the two cohorts due to the government’s restrictions on entering a school setting during the pandemic. In school, more distance between students and school staff was necessary; and research assistants were not allowed to enter the schools. Thus, in Cohort 2, there was no group testing in school. Questionnaires were filled out at home with parents, and parents were instructed how to go through the questionnaires with their children. We cannot rule out the possibility that the parents’ presence consciously or unconsciously impacted their children’s responses. However, filling out this questionnaire was a no-stakes situation because students were asked about their intrinsic value (personal interest) and self-concept (belief that they could solve certain tasks) related to specific academic tasks. Thus, we did not expect a specific bias due to parental involvement. Fourth, the study followed a cohort-control design with pretest. Thus, only observable confounders (i.e., alternative factors explaining the differences between the two cohorts) could have been controlled for in this study. Nevertheless, we assume that, for our investigation of the effects of learning during the pandemic on students’ achievement and motivation, the most relevant confounders were initial achievement, prior motivation, and students’ background. Even though single unobserved confounders might exist, strong relationships between these confounders and the outcomes as well as between the confounders and the cohorts would be necessary to diminish the observed results (see the findings from the sensitivity analysis, Table S1). Furthermore, we ruled out any history effects in addition to the pandemic because the assessments of two cohorts were spaced only one year apart. Thus, given the circumstances, we had a strong design for investigating the effects of the pandemic on students’ learning-related outcomes in different schools. Fifth, teacher-student contact was used as a proxy for (individual) student support. We measured how often teachers had contact with a child or parent to help them complete the assignments or provide feedback. We had no information about the quality of support (positive encouragement vs. emotional support vs. academic feedback), form of support (phone calls, online meetings, high-quality written feedback), or responsiveness or pertinence (whether it met individual needs). We only had information about the frequency of teacher-student contact. However, the contact between teachers and students itself is a pre-condition for all support practices. Therefore, we might have even underestimated the influence of (individual) student support in our study. Finally, the existing baseline differences in reading achievement between the two cohorts might have obscured a potential negative effect of the pandemic on reading achievement at the end of Grade 1. And the initial difference in motivation related to language activities might be associated with an underestimation of the actual negative effects of the pandemic on students’ self-concept and intrinsic value in reading at the end of Grade 1. However, these implications are speculative in nature, and we controlled for these variables in our analyses.

Overall, we found evidence for the importance of teacher-student contact in the first wave of the pandemic—at the beginning of compulsory schooling. Although we did not observe negative effects of pandemic-related school closures on math and reading achievement, we found that high teacher-student contact was advantageous for learning during the pandemic (vs. regular schooling) for math achievement. Furthermore, we obtained a rare and important insight into the negative consequences of the first wave of the pandemic on students’ motivation in writing and reading.

## Methods

### Sample and procedure

The data stemmed from a randomized controlled field study conducted in the German state of Baden-Württemberg that has started in the 2018/2019 school year (FIPS+ study). The FIPS+ study was originally designed to assess the implementation of a school-entry assessment measuring vocabulary, phonological awareness, reading capabilities, mathematical abilities, and general cognitive ability in school right at the beginning of Grade 1 (the beginning of formal schooling). On the basis of the findings from an a priori power analysis, we aimed to recruit 200 schools that would begin the study in September 2018. However, only 88 schools registered to participate (Cohort 1). Thus, we also advertised the study during the following year, and 75 new schools registered to start in September 2019 (Cohort 2), and 19 schools from Cohort 1 continued to participate. Cohort 1 comprised 1,405 students in 88 classes; and Cohort 2 comprised 1,335 students in 94 classes. Due to the start of the pandemic in the 2019/2020 school year, we were able to use this cohort-control design with pretest^45^ to estimate the effects of the COVID-19-related school closures. For further details related to the procedure see Note S3. The final sample consisted of 2,740 students, 2,248 parents, 245 teachers, and 163 school principals (see Table 1).

The sample was a convenience sample with the following characteristics compared with the population of primary-school classes in Baden-Württemberg: On average, the class sizes were similar to typical class sizes (*M*_Sample_ = 18.97 vs. *M*_Population_ = 19.80), and the percentage of girls (vs. boys) was approximately representative with slightly more boys than girls (*M*_Sample_ = 49.6% girls and *M*_Population_ = 49.3% girls); there were fewer children who did not speak German with their parents (*M*_Sample_ = 19.11%, *M*_Population_ = 23.25%); and the proportion of students with a migration background was smaller (*M*_Sample_ = 26.35%, *M*_Population_ = 30.46%); the socioeconomic status (SES) of the families could not be compared with the state level; however, students in Baden-Württemberg tend to come from families with slightly higher HISEI scores compared with the national average^46^, and the HISEI score in our sample was also slightly higher than the national average if we compared it with available monitoring data from fourth-grade students from the IQB-Bildungstrend 2021 (*M*_IQB2021_ = 51.50; *M*_Sample_ = 58.21; up to 1/3 SD more). See Table 2 for additional details.

### Ethics

This study was approved by the ethics committee of the DIPF | Leibniz Institute for Research and Information in Education in Frankfurt (preliminary approval was granted on September 04, 2018; final approval was granted on October 19, 2018) and the Ministry of Education, Youth, and Sport in Baden-Württemberg (approved on May 28, 2018, reference number: 31-6499.20/1191). Due to the pandemic, the questionnaires that had originally been planned for parents and teachers were adapted and extended to be administered to Cohort 2. These adaptions were also approved by both institutions (ethics committee: June 4, 2020; Ministry of Education, Youth, and Sport in Baden-Württemberg: May 15, 2020).

### Demographics

Teachers provided information on student gender (0 = male, 1 = female) and date of birth. Migration background was coded on the basis of parent reports of the countries in which each parent and the child were born. If the mother, father, or child was born outside Germany, the student had a migration background (1 = yes, 0 = no). The SES of a child’s family was measured with the Highest International Socio-Economic Index of Occupational Status (HISEI) with respect to the parents’ current occupations. The HISEI represents the score of the parent with the highest International Socio-Economic Index of Occupational Status, ISEI^47^, calculated in line with the International Standard Classification of Occupations 2008, ISCO-08^48^.

### Students’ school achievement

We used a test called FIPS+, which consisted of a combination of two validated instruments, and we administered them on tablet computers: a) German translation and adaptation of the Performance Indicators in Primary School (PIPS)^49–51^ called Fähigkeitsindikatoren Primarschule (FIPS; Capability Indicators in Primary School)^52,53^ and b) a nonverbal intelligence subscale from the BUEGA^54,55^, a German test that is widely used to identify developmental disorders in primary-school-aged children. Both are standardized measurement instruments, administered in individual test settings, adaptive, and appropriate for both measurement occasions for this age group. The total testing time took between 20 and 40 min. For reading achievement, scores could range from 0 to 159 points; for math achievement, from 0 to 53 points; and for the general cognitive task, from 0 to 38 points. The internal consistency scores at the beginning of Grade 1 were: 0.98 (reading achievement), 0.92 (math achievement), and 0.88 (general cognitive task). The internal consistency scores at the end of Grade 1 were: 0.95 (reading achievement), 0.91 (math achievement), and 0.89 (general cognitive task). For further details, see Tables 3 to 5. As suggested by the test manuals, we used sum scores for further analyses. In this study, we focused on reading and math achievement as outcome variables. Due to a technical problem with the computer-based assessment system in Cohort 1, some items were not administered to some students who should have answered these items. For these children, we had to estimate the missing scores on the reading tasks on the basis of their response patterns on the previous tasks. In the Supplementary Information, we provide a complete overview of the data, including descriptive statistics as well as the results of the main analyses in which we demonstrate that the procedure that was chosen to handle the technical problem seems to be appropriate (see Note S4 and Tables S17–S21).

### Students’ motivation

We focused on students’ academic self-concepts and students’ interests as two particularly important aspects of motivation^16,56^. To assess domain-specific math and verbal self-concepts and individual (or personal) interests, we used an adapted version of the Self Description Questionnaire for preadolescents (SDQ)^57^ and an adaptation of the Self Description Questionnaire for preschoolers (SDQP)^58^. At the beginning of Grade 1, we asked about typical activities related to math or language such as “I enjoy playing with numbers” or “I enjoy listening to stories” and “I enjoy looking at books.” We could not ask questions that were directly related to school subjects because the students were not necessarily familiar with math and German. The internal consistencies were 0.57 (math) and 0.54 (language). At the end of Grade 1, we asked students about their domain-specific self-concepts and interests in reading, writing, and math tasks by having them rate items such as “I am good at reading” and “Reading is easy for me” or “I enjoy calculating.” The internal consistencies were appropriate for math (interest: 0.86, self-concept: 0.85), reading (interest: 0.88, self-concept: 0.84), and writing (interest: 0.88, self-concept: 0.81). Each item was rated on a 3-point scale with smileys representing the values 1 (always true), 2 (sometimes true), and 3 (not true). We recoded all items to ensure that high scores indicate high motivation. We used mean scores for further analyses.

### School and class characteristics

To assess characteristics of the schools, we administered a questionnaire to the school principals at the beginning of Grade 1. For this study, we used the proportion of students with a migration background as a school-level variable because previous research has suggested an association between the proportion of students with a migration background in school on educational outcomes such as grade repetition and track attendance^29^. The school principals were asked to provide the exact numbers. On the class level, the size of each class was provided by the teacher and ranged from 4 to 30 students. We used class size because a recent study suggested an advantage of smaller class sizes on students’ achievement during the pandemic^28^. Thus, we used class size and proportion of students with a migration background as control variables in our analyses because we expected that these variables might have affected teaching during the pandemic.

### Treatment moderator—contact between teachers and students

In Cohort 2, the teachers were asked to use a 5-point rating scale to report how often they had contact with each student in their class or the parents of this student. The question was “How often did you have contact with the child or parent to help them complete the assignments or provide feedback?” The response format was 1 (almost every day), 2 (several times per week), 3 (once per week), 4 (occasionally), and 5 (never). The content and response format are comparable to the ones used in a representative German study when the schools first closed^23^. Overall, the information about class teachers’ contact with their students was available for 609 students in Cohort 2 at the end of Grade 1. Only 26 students had contact with their teachers almost every day, 123 had contact several times per week, 296 had contact once per week, 125 had contact occasionally, and only 29 had no contact at all.

This question was not posed to Cohort 1, which comprised students who attended school under regular learning conditions in Grade 1 before the pandemic. Because this information was missing for Cohort 1, we could not include a term that represented the interaction between treatment (i.e., cohort) and teacher-student contact in our analyses. Furthermore, due to the small sample sizes in the extreme categories, we decided to combine categories in such a way that would lead to a meaningful contrast. Therefore, we differentiated between three teacher-student contact conditions in the subsequent analyses: 0 = never or occasionally, 1 = once per week, and 2 = several times per week or almost every day. This distribution of contact matched the findings by Wößmann et al. ^23^. They found that most teachers seemed to be able to provide learning materials, feedback, or individual teacher-student contact (e.g., providing learning sheets was most common, and individual conversations and teaching the whole class via video calls were most rare) at least once per week. Thus, we assumed that teacher-student contact that occurred less frequently than once per week represented the most critical situation during the school closures and was likely associated with the largest learning losses. Contact once per week reflected the modal and, under pandemic conditions, arguably the most realistic form of instructional support, for which negative effects could still be expected but potentially to a smaller extent. Finally, more frequent contact (several times per week or almost daily) constituted a particularly relevant condition, as it may be associated with smaller learning impairments or even null or positive effects on students’ outcomes.

### Baseline equivalence

To test whether baseline differences existed and might bias the estimates of the effects, we ran a regression model in which all baseline measures were predicted by the cohort variable. We used Mplus 8.5^59^ to conduct the analysis. To account for potentially nonnormally distributed variables and the multilevel structure of the data (students nested in classes), we used cluster-robust standard errors^60^. To handle missing values, we used full information maximum likelihood (FIML) estimation. Results are presented in Table 3.

### Drop-out in Cohort 2

To test whether drop-out in Cohort 2 could be predicted by any pretest measure, we computed a logistic regression analysis. We used Mplus 8.5^59^ to conduct the analysis. To account for potentially nonnormally distributed variables and the multilevel structure of the data (students nested in classes), we used cluster-robust standard errors^60^. To handle missing values, we used full information maximum likelihood (FIML) estimation. Table 4 presents the results.

### Analyses

To address RQs 1 to 3, we used Mplus 8.5^59^ to compute multiple linear regression analyses. To account for potentially nonnormally distributed variables and the multilevel structure of the data (students nested in classes), we used cluster-robust standard errors^60^. To handle missing values, we used full information maximum likelihood (FIML) estimation. We followed an analysis plan consisting of two steps to (a) estimate the effects of learning under pandemic conditions (and the extent to which the heterogeneity in these effects depended on students’ family SES, baseline performance, or baseline motivation) and (b) estimate the effects of the potential treatment moderator (and the extent to which the heterogeneity in these effects depended on students’ family SES, baseline performance, or baseline motivation).

We ran two sets of models, each including a three-step procedure. In the first set of models, the treatment variable was cohort (0 = Cohort 1 and 1 = Cohort 2). In the second set of models, we split the treatment condition *learning during the pandemic* into three subconditions: *learning during the pandemic with low teacher-student contact*, *learning during the pandemic with medium teacher–student contact*, and *learning during the pandemic with high teacher-student contact*. We built three indicator variables as treatment variables and used all three to predict the target outcomes. For each indicator, the reference category was regular learning (i.e., Cohort 1). Thus, Indicator 1 equaled 1 for students in Cohort 2 with low contact between students and teachers (i.e., occasional or no contact) and 0 otherwise. Indicator 2 equaled 1 for students in Cohort 2 with medium contact between students and teachers (i.e., once a week) and 0 otherwise. Indicator 3 equaled 1 for students in Cohort 2 with high contact between students and teachers (i.e., several times per week or almost every day) and 0 otherwise. We computed a power analysis (conducted with PowerUpR^61^) for a two-level RCT with the treatment on Level 2 (as the most conservative approach for estimating the minimum detectable effect size in this study), power = 0.80, alpha = 0.05 (two-tailed), number of Level 2 covariates = 2, ICC = 0.10, proportion of variance in the outcome explained by Level 2 covariates = 0.10, average number of students in a class = 15, number of classes = 132, and variability in the proportions of variance in the outcome explained by the Level 1 covariates. The results of the power analysis showed that the sample size in this study was appropriate to detect effects of the pandemic ranging from *d* = 0.19 to d = 0.26 in the low- and high-contact conditions that were as strong as the effect sizes reported in the meta-analyses by Storey and Zhang^1^, Betthäuser et al.^2^, and Di Pietro^3^: *d* = −0.21, *d* = −0.14, and *d* = −0.17, respectively.

In the first step, all baseline measures as well as the treatment variable(s) were included as predictor variables to predict achievement and the motivational variables at the end of Grade 1. In the second step, we additionally included the interactions between students’ family SES and the treatment variable(s) in the model to investigate whether potential treatment effects were moderated by students’ family SES. In the third step, we included the interactions between students’ pretest scores on the corresponding outcome variable and the treatment variable(s) in the model—instead of the interaction between students’ family SES and the treatment variable(s)—to estimate whether students with low initial achievement or motivation scores were at a greater disadvantage than their peers. By using the Wald chi-square test implemented in Mplus, we could compare the effect of the school closures for students with low contact with their teachers compared with regular schooling with the effect of the school closures for students with medium or high contact with their teachers compared with regular schooling. To do this, we used the model constraint command in Mplus^62^. For additional technical details (i.e., equations) related to the estimates in this study, see Estimation Strategy.

To do robustness checks, we controlled for different sources of variance and followed all the steps of the analyses for different subsamples. First, we ran the analyses with all the cases in our sample. Second, we ran the analyses only with the cases that remained in our sample in Cohort 2 at the second measurement occasion. Third, we ran the analyses only for the experimental manipulation in which teachers conducted the school-entry assessment with their students. Fourth, we ran the analyses only with the schools that had students in both cohorts. In doing so, we controlled for different sources of variance to estimate the effect of learning during the pandemic. In the main text, we report the results for the complete sample and only for the subsamples if any meaningful differences in the patterns of results were observed. All results from the additional analyses are reported in the Supplementary Information (Tables S2– S16).

### Estimation strategy

To address Research Questions 1 and 2, we used the following equations:

Estimation strategy – without interaction terms:

1 $$Y=\alpha +\beta X+{\sum }_{m=1}^{M}{Z}_{m}$$

Y: student outcome

X: treatment condition (0 = Cohort 1, 1 = Cohort 2)

Z: covariate

α: intercept

β: average effect of learning during the pandemic

Estimation strategy – with interaction terms (SES and baseline score):

2 $$Y=\alpha +{\beta }_{1}X+{\beta }_{2}{SES}+{\beta }_{3}X* {SES}+{\sum }_{m=1}^{M}{Z}_{m}$$

${\beta }_{1}:$ average effect of learning during the pandemic if SES was equal to 0 (i.e., average SES)

${\beta }_{3}$ : average effect of learning during the pandemic if SES was not equal to 0

To address Research Question 3, we used the following equations:

Estimation strategy – without interaction terms:

3 $$Y=\alpha {+\beta }_{1}{X}_{1}{+\beta }_{2}{X}_{2}{+\beta }_{3}{X}_{3}+{\sum }_{m=1}^{M}{Z}_{m}$$

${X}_{1}:\,$ treatment condition 1 (1=Cohort 2 and low teacher-student contact, 0=else)

${X}_{2}:\,$ treatment condition 2 (1=Cohort 2 and medium teacher-student contact, 0=else)

${X}_{3}:\,$ treatment condition 3 (1=Cohort 2 and high teacher-student contact, 0=else)

${\beta }_{1}:$ average effect of learning during the pandemic if teacher-student contact was low

${\beta }_{2}:$ average effect of learning during the pandemic if teacher-student contact was medium

${\beta }_{3}:$ average effect of learning during the pandemic if teacher-student contact was high

Estimation strategy – with interaction terms (SES and baseline score):

4 $$Y=\alpha {+\beta }_{1}{X}_{1}{+\beta }_{2}{X}_{2}{+\beta }_{3}{X}_{3}+{\beta }_{3}{SES}{+\beta }_{4}{X}_{1}{SES}+{\beta }_{5}{X}_{2}{SES}{+\beta }_{6}{X}_{3}{SES}{\sum }_{m=1}^{M}{Z}_{m}$$

${\beta }_{1}:$ average effect of learning during the pandemic if teacher-student contact was low (if SES was equal to 0)

${\beta }_{2}:$ average effect of learning during the pandemic if teacher-student contact was medium (if SES was equal to 0)

${\beta }_{3}:$ average effect of learning during the pandemic if teacher-student contact was high (if SES was equal to 0)

${\beta }_{4}:$ average effect of learning during the pandemic if teacher-student contact was low (if SES was not equal to 0)

${\beta }_{5}:$ average effect of learning during the pandemic if teacher-student contact was medium (if SES was not equal to 0)

${\beta }_{6}:$ average effect of learning during the pandemic if teacher-student contact was high (if SES was not equal to 0)

## Supplementary information

SI_R1_2026Feb.

## Data Availability

The dataset generated and/or analyzed during the current study are not publicly available due to their inclusion in an ongoing study at the Hector Research Institute of Education Sciences but are available from the corresponding author on reasonable request.

## Code availability

Our custom code is available in the Open Science Framework repository at https://osf.io/gbx6w/overview?view_only=0424da03366c4554bdcaa77ca9873b03. We used Mplus 8.5 for the final analyses.
