# Supplementary material for: COVID-19-related school closures and students’ achievement and motivation: Did teacher support make a difference
Source: NPJ Sci Learn. 2026 Aug 19;11:48. doi: 10.1038/s41539-026-00439-1 (PMC13490548; doi:10.1038/s41539-026-00439-1)
Supplement: Supplementary file 1 [file 41539_2026_439_MOESM1_ESM.pdf]

Supplementary Information

Note S1. The Situation in Germany During the First Lockdown

In Germany, schools closed completely for an average of 5 weeks and partially for 10 weeks between March and August 2020 (UNESCO, 2022). As in other OECD countries, the federal states of Germany reacted quickly to the unforeseen situation, but the schools had to improvise to ensure that learning continued. According to representative teacher reports in April 2020 (in the middle of the first lockdown), almost all schools provided learning materials during the lockdown, but only a few schools had already used concepts of remote teaching or were at least familiar with them. The majority of teachers reported that they were left on their own (Forsa, 2020; Vodafone Stiftung Deutschland, 2020).

Learning was dramatically impaired during the school closures in the first half of 2020. The total amount of time students spent on schoolwork was cut in half (Wößmann et al., 2020), and learning consisted of students working at home alone or with their parents on exercise sheets, reading texts, watching learning videos, and using educational software or programs. Lessons with all students present in one class simultaneously via video conferences were rarely reported, and individual contact between teachers and students also occurred only rarely (Vodafone Stiftung Deutschland, 2020; Wößmann et al., 2020). Parents reported several problems with remote learning, such as a lack of support from schools, a lack of digital competencies in teachers, misleading tasks, too many and poorly coordinated communication channels, problems with software being too complex or difficult to use, as well as legal issues (e.g., data security), and even problems with connectivity and too few or old devices (Initiative D21 & TU München, 2020). Almost all parents reported that the schools assigned a schoolwork packet at least once a week, and the majority of students returned it to teachers at least once a week (Wößmann et al., 2020). However, only half of the parents reported that learning materials were processed daily, and less than one third of students turned work in to teachers on a daily basis. Teachers provided feedback—at least once a week—in more than half of the reports (according to parents). In addition, students from families with low SES and students with low school achievement suffered from more disadvantages than their peers (Wößmann et al., 2020).

Reports from this time period that differentiated between primary and secondary schools revealed that, for German primary school students, learning was even more affected by the pandemic than it was for secondary school students. For instance, teachers in primary schools provided a smaller total amount of learning materials irrespective of the format (i.e., print or digital) than teachers in secondary schools did (Vodafone Stiftung Deutschland, 2020). Primary school teachers felt less prepared for the new situation and less often reported that the schools had an overarching concept that allowed them to react appropriately to the new challenges compared with secondary school teachers. Primary-school-aged students and their parents received the learning materials either in a printed format before the school closures or in a printed or digital format during the school closures and almost no instructions via video conferences (Huebener et al., 2020). Furthermore, overall, primary school students spent less time on school-related activities compared with students in secondary school. But, in contrast to students in secondary school, primary school students spent more time on activities such as

reading, creative activities, and exercises, and less time playing games on computers, mobile phones, or game console than older students did (Wößmann et al., 2020).

Note S2. The Situation for Primary School Students in Baden-Württemberg During the First Lockdown

Each of Germany's 16 states has its own Ministry of Education, which administers and governs educational matters independently. About 13% of the German population lives in the state of Baden-Württemberg. In a joint decision in early March, the federal ministries decided to close schools within 1 week. Schools in Baden-Württemberg were closed on March 17, 2020. Primary schools in Baden-Württemberg began reopening on May 18, but for Grades 1, 2, and 3, in-person teaching was resumed June 15 if possible. Some schools continued hybrid teaching with weekly changes between in-person learning and remote learning until summer vacations began on July 30. All in all, first-graders missed between 9 and 12 weeks of in-person learning during the first wave of the pandemic in 2020 (not counting 4 weeks of regular school holidays). Teachers mainly provided paper worksheets and learning packages before the full lockdown was enacted. Initially, the focus was on rehearsing previously learned content. Four weeks into the school lockdown, the ministry recommended that teachers provide worksheets and textbook tasks for students at home. It remained optional to use digital tools to administer remote teaching on a regular basis during the school closures. A survey of parents (n = 194) and teachers (n = 121) in Baden-Württemberg found that keeping students motivated to learn was a bigger challenge than technical issues related to online teaching (Immerfall, 2021).

Note S3. Procedure

In this study, we focused on the first two measurement occasions at the beginning and end of Grade 1 in both cohorts(Cohort 1: 2018/2019 school year, Cohort 2: 2019/2020 school year). At the beginning of Grade 1, teachers or research assistants conducted individual student testing, including the school-readiness assessment as well as an interview-like survey containing questions related to the school-readiness assessment and students' motivation to engage in academic activities. At the end of Grade 1, the administration of tests and questionnaires differed slightly between the two cohorts. In Cohort 1, as planned, teachers or research assistants conducted the end-of-the-year assessment individually and sitting directly next to the children 2–5 weeks before the school year ended. During this period, there was also a group-testing situation in which research assistants administered a paper-pencil survey lasting 45 min to the students in every class. The survey included motivational variables as well as information pertaining to teaching and instruction in German and mathematics classes during that school year. In Cohort 2, the research assistants were not allowed to enter the schools, and it was necessary to keep a distance of 1.5 m between the test administrators and the children at the end of the year due to the pandemic. Thus, school staff (i.e., teachers, interns, social workers) conducted the end-of-the-year assessment individually with the students on a tablet computer and with instructions to maintain a distance of 1.5 m between the students and the school staff. A second computer screen and an additional mouse were used. Because no group testing was allowed in the schools, the additional questionnaires were sent out to the families. Parents were instructed to fill out the questionnaire with their children. To create a comparable situation for all students, parents were specifically taught how to complete the questionnaire with the children and to read all items aloud step by step. The questionnaires were sent to the families at the beginning of the 8th week before summer vacation and were collected and sent back by

the teachers. The administration of this study was conducted by an external institution (IEA Hamburg, Link: https://www.iea.nl/). All teachers, school staff, and research assistants were trained and had experience working with primary school students before the study began.

Note S4. Technical Problem – Reading Measure

Due to a technical problem in Cohort 1, the most difficult reading tasks were not presented to some children who showed a specific response pattern. Thus, the missing data pattern was not at random, and common procedures (e.g., multiple imputation) could not be applied to replace these missing values. For these children, we estimated the data points in the reading tasks by predicting the data points on the basis of the children's response patterns in the previous tasks. The technical problem was a programming error that occurred after adapting the FIPS to the FIPS+. We solved this problem before testing Cohort 2. Here, we provide a complete overview of the data, including descriptive statistics as well as the results of the main analyses in which we used four different methods to handle missing data in the reading tasks. These methods included: (a) using the predicted scores as we did in the main part of the manuscript, (b) using NAs on the sum score instead of predicted values, (c) using the minimum possible reading score for the tasks (i.e., items) that should have been presented but were not presented, and (d) using the maximum possible reading score for the tasks (i.e., items) that should have been presented but were not presented. Tables S17 to S21 present all the results. We found differences across these approaches in the descriptive statistics and baseline measures (Tables S17 and S18). However, the patterns in the findings on the effects of the pandemic were comparable across three out of the four missing-data approaches (Tables S19 to S21). The findings were comparable for (a), (b) and (d), indicating that the most difficult items were not presented to some of the children. Therefore, our chosen procedure—to estimate missing values on an item level for these children—seems to be appropriate.

Figure S1. Histogram of Reading Achievement at the End of Grade 1

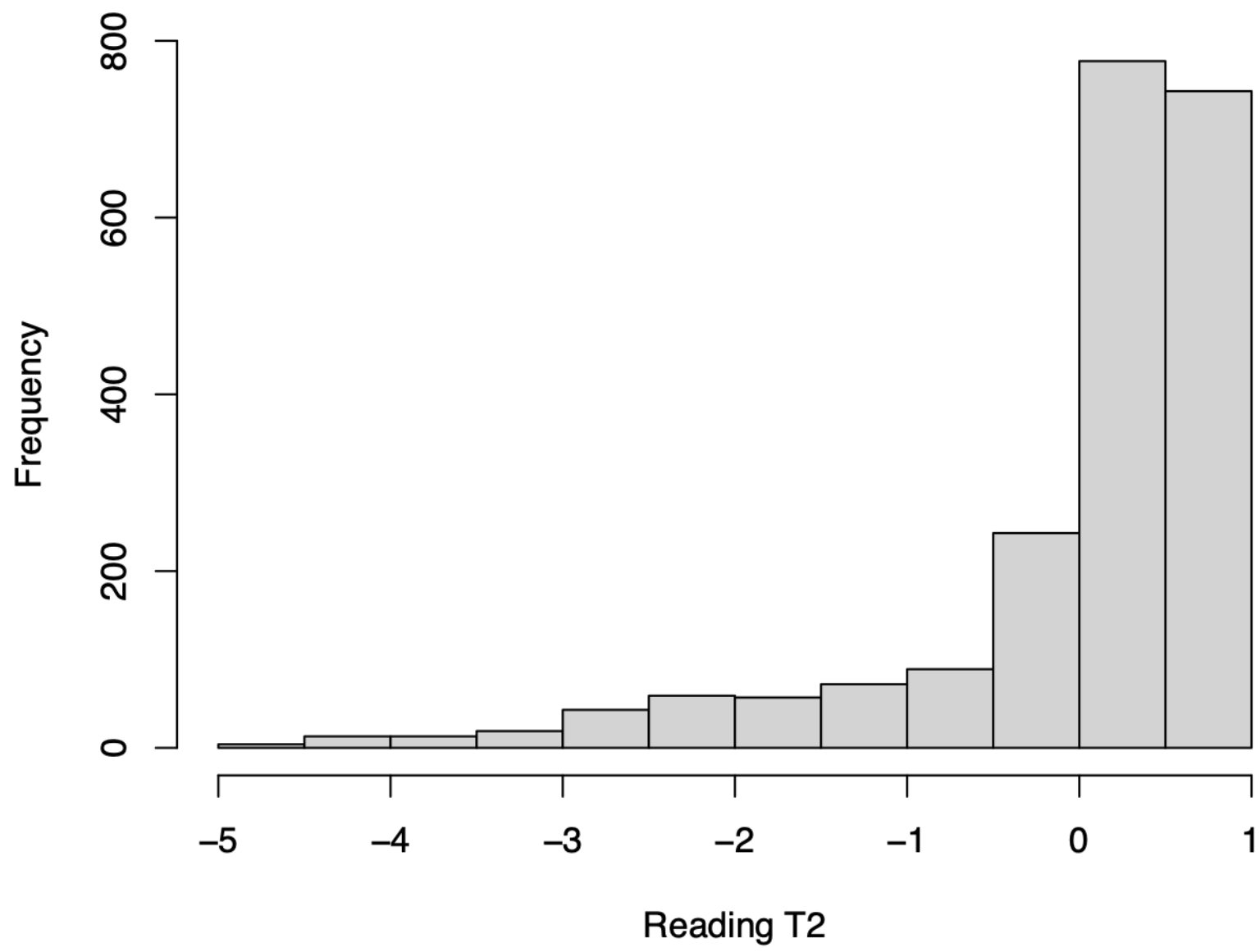

Sensitivity Analysis

Table S1
Results of the Sensitivity Analysis

Dependent variables	Multiple regression coefficient (ES in SD_Y)	Effect of the unobserved confounder on the outcome (δ)		
		0.10	0.30	0.50
Achievement				
Reading	0.03	0.30	0.10	0.06
Mathematics	-0.01	-0.10	-0.03	-0.02
Motivational variables				
Intrinsic value				
Mathematics	0.04	0.40	0.13	0.08
Reading	-0.12	-1.20	-0.40	-0.24
Writing	-0.15	-1.50	-0.50	-0.30
Self-concept				
Mathematics	0.01	0.10	0.03	0.02
Reading	-0.14	-1.40	-0.47	-0.28
Writing	-0.17	-1.70	-0.57	-0.34

Note. We conducted a sensitivity analysis to assess the robustness of our findings in the presence of single, unobserved confounding variables (Foster, 2010; VanderWeele & Arah, 2011). We calculated the difference that would need to occur in the possible confounder between the treatment and the control groups to eliminate the previously observed findings (for similar applications, see Hogrebe & Strietholt, 2016; Wagner, Becker, Lüdtke, & Trautwein, 2015). VanderWeele and Arah (2011) proposed the following formula to assess the amount of bias in the presence of an unobserved confounder: $bias = \gamma * \delta$. The bias is a function of the relationships between the confounder and the outcome (δ) and the confounder and the treatment (γ). To figure out the magnitude of the difference that would need to occur in the possible confounder between the treatment and the control groups to eliminate the previously observed findings (γ), we need to rearrange the formula, $\gamma = bias/\delta$. If the effect on reading interest is just a bias (bias = -0.12), and an unobserved confounder (in addition to the covariates we considered) has an effect of 0.30 on the outcome (δ = 0.30), the difference that would need to be found in the confounder between the treatment and the control groups must be -0.40 SD (γ = -0.12 / 0.30). An unobserved confounder with a small effect (ES = 0.10) on reading interest could diminish the effect of the pandemic-related school closures on reading interest if the difference between Cohort 2 and Cohort 1 is -1.20 SD (γ = -0.12 / 0.10) in the unobserved confounder. A confounder with a large effect on the outcome (δ = 0.50) would have to create a -0.24 SD difference between the two groups to eliminate the effect of the pandemic-related school closures on reading interest (γ = -0.12 / 0.50).

Results for Three Different Subsamples

Only Cases That Remained in the Sample in Cohort 2 (N = 2,278)

Table S2. Effects of the COVID-19 Pandemic

Variables	Multiple regression coefficient (ES in SD_Y)	*SE*	*z*	*p*
Achievement				
Reading	0.02	0.07	0.35	.726
Mathematics	|<0.01|	0.05	-0.08	.940
Motivational variables				
Intrinsic value				
Mathematics	0.08	0.05	1.51	.131
Reading	-0.10	0.06	-1.76	.079
Writing	-0.15	0.05	-2.98	.003
Self-concept				
Mathematics	0.05	0.05	1.00	.318
Reading	-0.13	0.05	-2.48	.013
Writing	-0.17	0.05	-3.34	.001

Note. The table presents the regression coefficients for cohort (treatment variable) in a multiple linear regression analysis in which all dependent variables were considered simultaneously and all pretest measures were used as control variables. We ran the analysis in Mplus 8.5 (Muthén & Muthén, 1998–2017). To account for potentially nonnormally distributed variables and the multilevel structure of the data (students nested in classes), we used cluster-robust standard errors (for a justification, see McNeish et al., 2017). To handle missing values, we used full information maximum likelihood (FIML) estimation. All variables were z-standardized before the analysis except for the dichotomous variables (cohort, gender, and migration background). Thus, the regression coefficients can be interpreted as effect sizes.

Only Cases That Remained in the Sample in Cohort 2 (N = 2,278)

Table S3. Effects of the COVID-19 Pandemic – Moderated by HISEI

Variables	Cohort effect (ES in SD_Y)				HISEI (ES in SD_Y)				Interaction term Cohort x HISEI (ES in SD_Y)			
	b	*SE*	*z*	*p*	*b*	*SE*	*z*	*p*	*b*	*SE*	*z*	*p*
Achievement												
Reading	0.02	0.07	0.35	.726	0.12	0.03	4.01	<.001	-	-	-	-
Mathematics	|<0.01|	0.05	-0.07	.944	0.04	0.03	1.83	.067	-0.01	0.04	-0.16	.869
Motivational variables												
Intrinsic value												
Mathematics	0.08	0.05	1.51	.131	-0.10	0.03	-2.90	.004	0.01	0.05	0.19	.852
Reading	-0.10	0.06	-1.79	.073	0.07	0.03	2.00	.045	0.10	0.06	1.71	.087
Writing	-0.15	0.05	-3.02	.003	-0.03	0.04	-0.71	.479	0.07	0.05	1.43	.154
Self-concept												
Mathematics	0.05	0.05	0.99	.320	-0.06	0.04	-1.68	.092	|<0.01|	0.05	0.09	.925
Reading	-0.13	0.05	-2.53	.012	0.07	0.03	2.65	.008	0.11	0.06	2.09	.036
Writing	-0.17	0.05	-3.33	.001	0.06	0.03	1.88	.060	-0.02	0.06	-0.38	.702

Note. The table presents the regression coefficients for cohort (treatment variable), HISEI score, and the interaction between cohort and HISEI score in a multiple linear regression analysis in which all dependent variables were considered simultaneously and all pretest measures were used as control variables. We ran the analysis in Mplus 8.5 (Muthén & Muthén, 1998–2017). To account for potentially nonnormally distributed variables and the multilevel structure of the data (students nested in classes), we used cluster-robust standard errors (for a justification, see McNeish et al., 2017). To handle missing values, we used full information maximum likelihood (FIML) estimation. All variables were z-standardized before the analysis except for the dichotomous variables (cohort, gender, and migration background). Thus, the regression coefficients can be interpreted as effect sizes.

Only Cases That Remained in the Sample in Cohort 2 (N = 2,278)

Table S4. Effects of the COVID-19 Pandemic – Moderated by Baseline Level

Variables	Cohort effect (ES in SD_Y)				Pretest (ES in SD_Y)				Interaction term Cohort x Pretest (ES in SD_Y)			
	b	*SE*	*z*	*p*	*b*	*SE*	*z*	*p*	*b*	*SE*	*z*	*p*
Achievement												
Reading	0.02	0.07	0.34	.733	0.09	0.01	6.29	<.001	-	-	-	-
Mathematics	|<0.01|	0.05	-0.09	.930	0.58	0.03	20.52	<.001	0.01	0.04	0.27	.789
Motivational variables												
Intrinsic value												
Mathematics	0.07	0.05	1.44	.151	0.2	0.04	5.09	<.001	-0.10	0.05	-1.95	.051
Reading	-0.10	0.06	-1.83	.067	0.06	0.03	1.79	.073	0.06	0.05	1.23	.220
Writing	-0.15	0.05	-2.96	.003	0.06	0.03	1.80	.071	0.04	0.04	0.84	.404
Self-concept												
Mathematics	0.04	0.05	0.88	.376	0.15	0.04	3.73	<.001	-0.07	0.05	-1.51	.130
Reading	-0.14	0.05	-2.55	.011	0.08	0.03	2.58	.010	0.04	0.04	0.97	.332
Writing	-0.17	0.05	-3.29	.001	0.05	0.03	1.74	.082	0.03	0.05	0.63	.529

Note. The table presents the regression coefficients for cohort (treatment variable), pretest score, and the interaction between cohort and pretest score in a multiple linear regression analysis in which all dependent variables were considered simultaneously and all pretest measures were used as control variables. We ran the analysis in Mplus 8.5 (Muthén & Muthén, 1998–2017). To account for potentially nonnormally distributed variables and the multilevel structure of the data (students nested in classes), we used cluster-robust standard errors (for a justification, see McNeish et al., 2017). To handle missing values, we used full information maximum likelihood (FIML) estimation. All variables were z-standardized before the analysis except for the dichotomous variables (cohort, gender, and migration background). Thus, the regression coefficients can be interpreted as effect sizes.

Table S5. Effects of the COVID-19 Pandemic for Different Levels of Contact Between Teachers and Students

Variables	Indicator 1 – low contact (ES in SD_Y) "occasionally, never"						Indicator 2 – medium contact (ES in SD_Y) "once a week"						Indicator 3 – high contact (ES in SD_Y) "several times per week, almost every day"					
		95% CI						95% CI						95% CI				
	b	low	up	*SE*	*z*	*p*	*b*	low	up	*SE*	*z*	*p*	*b*	low	up	*SE*	*z*	*p*
Achievement																		
Reading	-0.21	-0.50	0.09	0.15	-1.40	.163	0.06	-0.10	0.22	0.08	0.70	.481	0.14	-0.13	0.41	0.14	1.00	.317
Mathematics	-0.11	-0.31	0.09	0.10	-1.10	.272	-0.01	-0.11	0.10	0.06	-0.11	.917	0.16	0.03	0.30	0.07	2.38	.017
Motivational variables																		
Intrinsic value																		
Mathematics	0.11	-0.11	0.34	0.12	0.97	.333	0.11	-0.03	0.26	0.07	1.54	.123	-0.09	-0.28	0.10	0.09	-0.95	.342
Reading	-0.22	-0.38	-0.06	0.08	-2.68	.007	0.02	-0.12	0.17	0.07	0.31	.754	-0.14	-0.36	0.07	0.11	-1.29	.196
Writing	-0.18	-0.36	0.01	0.09	-1.88	.060	-0.13	-0.29	0.02	0.08	-1.67	.096	-0.13	-0.30	0.04	0.09	-1.51	.132
Self-concept																		
Mathematics	0.02	-0.23	0.26	0.12	0.14	.893	0.07	-0.08	0.22	0.08	0.94	.348	-0.01	-0.19	0.17	0.09	-0.11	.911
Reading	-0.30	-0.46	-0.14	0.08	-3.60	.001	0.00	-0.15	0.15	0.08	0.04	.967	-0.22	-0.42	-0.02	0.10	-2.13	.033
Writing	-0.23	-0.41	-0.06	0.09	-2.62	.009	-0.08	-0.24	0.08	0.08	-1.02	.308	-0.19	-0.38	0.00	0.10	-1.96	.050

Only Cases That Remained in the Sample in Cohort 2 (N = 2,278)

Table S6. Differences Between the Effects of the COVID-19 Pandemic for Different Levels of Contact Between Teachers and Students

Variables	Difference Indicator 2 – Indicator 1 (ES in SD_Y) "once a week" vs. "occasionally or never"				Difference Indicator 3 – Indicator 2 (ES in SD_Y) "several times per week or almost every day" vs. "once a week"				Difference Indicator 3 – Indicator 1 (ES in SD_Y) "several times per week or almost every day" vs. "occasionally or never"			
	b	*SE*	*z*	*p*	*b*	*SE*	*z*	*p*	*b*	*SE*	*z*	*p*
Achievement												
Reading	0.27	0.15	1.76	.079	0.08	0.14	0.56	.573	0.35	0.20	1.77	.076
Mathematics	0.11	0.11	1.00	.318	0.17	0.08	2.16	.030	0.28	0.11	2.43	.015
Motivational variables												
Intrinsic value												
Mathematics	0.00	0.12	0.02	.981	-0.20	0.11	-1.87	.062	-0.20	0.15	-1.38	.168
Reading	0.24	0.10	2.47	.014	-0.17	0.12	-1.36	.172	0.07	0.13	0.57	.569
Writing	0.05	0.12	0.38	.703	0.00	0.11	-0.01	.995	0.05	0.13	0.36	.720
Self-concept												
Mathematics	0.05	0.13	0.41	.684	-0.08	0.12	-0.68	.496	-0.03	0.14	-0.19	.848
Reading	0.30	0.10	3.05	.002	-0.22	0.12	-1.79	.073	0.08	0.12	0.64	.522
Writing	0.15	0.11	1.40	.163	-0.11	0.12	-0.91	.365	0.04	0.13	0.34	.731

Group A (N = 1,371)

Table S7. Effects of the COVID-19 Pandemic

Variables	Multiple regression coefficient (ES in SD_Y)	*SE*	*z*	*p*
Achievement				
Reading	-0.05	0.10	-0.44	.658
Mathematics	-0.04	0.06	-0.56	.575
Motivational variables				
Intrinsic value				
Mathematics	0.07	0.07	0.95	.341
Reading	-0.12	0.07	-1.73	.085
Writing	-0.18	0.07	-2.57	.010
Self-concept				
Mathematics	0.04	0.06	0.64	.520
Reading	-0.22	0.07	-3.20	.001
Writing	-0.16	0.06	-2.54	.011

Note. The table presents the regression coefficients for cohort (treatment variable) in a multiple linear regression analysis in which all dependent variables were considered simultaneously and all pretest measures were used as control variables. We ran the analysis in Mplus 8.5 (Muthén & Muthén, 1998–2017). To account for potentially nonnormally distributed variables and the multilevel structure of the data (students nested in classes), we used cluster-robust standard errors (for a justification, see McNeish et al., 2017). To handle missing values, we used full information maximum likelihood (FIML) estimation. All variables were z-standardized before the analysis except for the dichotomous variables (cohort, gender, and migration background). Thus, the regression coefficients can be interpreted as effect sizes.

Group A (N = 1,371)

Table S8. Effects of the COVID-19 Pandemic – Moderated by HISEI

Variables	Cohort effect (ES in SD_Y)				HISEI (ES in SD_Y)				Interaction term Cohort x HISEI (ES in SD_Y)			
	b	*SE*	*z*	*p*	*b*	*SE*	*z*	*p*	*b*	*SE*	*z*	*p*
Achievement												
Reading	-0.05	0.11	-0.44	.660	0.11	0.04	2.50	.012	-	-	-	-
Mathematics	-0.04	0.06	-0.55	.584	0.03	0.03	0.89	.374	|<0.01|	0.06	-0.06	.952
Motivational variables												
Intrinsic value												
Mathematics	0.07	0.07	0.96	.337	-0.06	0.06	-1.15	.252	-0.01	0.07	-0.16	.872
Reading	-0.12	0.07	-1.78	.075	0.07	0.05	1.40	.160	0.12	0.08	1.52	.128
Writing	-0.18	0.07	-2.53	.012	0.03	0.05	0.57	.572	0.02	0.07	0.30	.765
Self-concept												
Mathematics	0.04	0.06	0.64	.521	-0.08	0.06	-1.32	.188	|<0.01|	0.07	0.05	.958
Reading	-0.22	0.07	-3.23	.001	0.11	0.04	2.73	.006	0.10	0.07	1.39	.164
Writing	-0.16	0.06	-2.5	.012	0.13	0.04	2.89	.004	-0.06	0.07	-0.77	.439

Note. The table presents the regression coefficients for cohort (treatment variable), HISEI score, and the interaction between cohort and HISEI score in a multiple linear regression analysis in which all dependent variables were considered simultaneously and all pretest measures were used as control variables. We ran the analysis in Mplus 8.5 (Muthén & Muthén, 1998–2017). To account for potentially nonnormally distributed variables and the multilevel structure of the data (students nested in classes), we used cluster-robust standard errors (for a justification, see McNeish et al., 2017). To handle missing values, we used full information maximum likelihood (FIML) estimation. All variables were z-standardized before the analysis except for the dichotomous variables (cohort, gender, and migration background). Thus, the regression coefficients can be interpreted as effect sizes.

Group A (N = 1,371)

Table S9. Effects of the COVID-19 Pandemic – Moderated by Baseline Level

Variables	Cohort effect (ES in SD_Y)				Pretest (ES in SD_Y)				Interaction term Cohort x Pretest (ES in SD_Y)			
	b	*SE*	*z*	*p*	*b*	*SE*	*z*	*p*	*b*	*SE*	*z*	*p*
Achievement												
Reading	-0.05	0.11	-0.44	.657	0.11	0.02	5.11	<.001	-	-	-	-
Mathematics	-0.04	0.06	-0.58	.563	0.57	0.04	13.75	<.001	0.04	0.06	0.64	.521
Motivational variables												
Intrinsic value												
Mathematics	0.06	0.07	0.93	.354	0.21	0.05	4.05	<.001	-0.14	0.07	-1.99	.046
Reading	-0.13	0.07	-1.85	.064	0.06	0.04	1.39	.163	0.12	0.06	1.97	.049
Writing	-0.18	0.07	-2.56	.010	0.06	0.04	1.61	.106	0.02	0.06	0.39	.697
Self-concept												
Mathematics	0.04	0.06	0.59	.558	0.15	0.05	3.02	.003	-0.05	0.06	-0.81	.415
Reading	-0.22	0.07	-3.20	.001	0.12	0.04	2.97	.003	0.03	0.06	0.52	.606
Writing	-0.16	0.06	-2.54	.011	0.08	0.04	1.99	.047	0.03	0.06	0.38	.701

Note. The table presents the regression coefficients for cohort (treatment variable), pretest score, and the interaction between cohort and pretest score in a multiple linear regression analysis in which all dependent variables were considered simultaneously and all pretest measures were used as control variables. We ran the analysis in Mplus 8.5 (Muthén & Muthén, 1998–2017). To account for potentially nonnormally distributed variables and the multilevel structure of the data (students nested in classes), we used cluster-robust standard errors (for a justification, see McNeish et al., 2017). To handle missing values, we used full information maximum likelihood (FIML) estimation. All variables were z-standardized before the analysis except for the dichotomous variables (cohort, gender, and migration background). Thus, the regression coefficients can be interpreted as effect sizes.

Table S10. Effects of the COVID-19 Pandemic for Different Levels of Contact Between Teachers and Students

Variables	Indicator 1 – low contact (ES in SD_Y) "occasionally, never"						Indicator 2 – medium contact (ES in SD_Y) "once a week"						Indicator 3 – high contact (ES in SD_Y) "several times per week, almost every day"					
		95% CI						95% CI										
	b	low	up	*SE*	*z*	*p*	*b*	low	up	*SE*	*z*	*p*	*b*	low	up	*SE*	*z*	*p*
Achievement																		
Reading	-0.32	-0.81	0.18	0.25	-1.25	.210	-0.05	-0.33	0.23	0.14	-0.36	.721	0.13	-0.20	0.46	0.17	0.78	.436
Mathematics	-0.17	-0.51	0.18	0.18	-0.93	.353	-0.01	-0.17	0.14	0.08	-0.18	.859	0.13	-0.04	0.30	0.09	1.52	.128
Motivational variables																		
Intrinsic value																		
Mathematics	0.21	-0.11	0.53	0.16	1.29	.197	0.07	-0.13	0.28	0.11	0.70	.482	-0.10	-0.34	0.13	0.12	-0.85	.396
Reading	-0.21	-0.45	0.03	0.12	-1.73	.083	-0.01	-0.23	0.21	0.11	-0.07	.944	-0.22	-0.50	0.07	0.15	-1.46	.144
Writing	-0.29	-0.53	-0.06	0.12	-2.43	.015	-0.21	-0.47	0.05	0.13	-1.56	.119	-0.18	-0.39	0.03	0.11	-1.71	.087
Self-concept																		
Mathematics	0.02	-0.35	0.40	0.19	0.13	.898	0.13	-0.10	0.36	0.12	1.14	.255	-0.05	-0.27	0.18	0.12	-0.40	.689
Reading	-0.37	-0.57	-0.17	0.10	-3.62	.001	-0.14	-0.37	0.10	0.12	-1.16	.245	-0.25	-0.51	0.01	0.13	-1.87	.061
Writing	-0.38	-0.66	-0.10	0.14	-2.68	.007	-0.12	-0.41	0.17	0.15	-0.79	.431	-0.17	-0.44	0.10	0.14	-1.24	.217

Group A (N = 1,371)

Table S11. Differences in the Effects of the COVID-19 Pandemic for Different Levels of Contact Between Teachers and Students

Variables	Difference Indicator 2 – Indicator 1 (ES in SD_Y) "once a week" vs. "occasionally or never"				Difference Indicator 3 – Indicator 2 (ES in SD_Y) "several times per week or almost every day" vs. "once a week"				Difference Indicator 3 – Indicator 1 (ES in SD_Y) "several times per week or almost every day" vs. "occasionally or never"			
	b	*SE*	*z*	*p*	*b*	*SE*	*z*	*p*	*b*	*SE*	*z*	*p*
Achievement												
Reading	0.27	0.25	1.06	.288	0.18	0.19	0.98	.328	0.45	0.28	1.58	.114
Mathematics	0.15	0.17	0.87	.385	0.15	0.11	1.37	.171	0.30	0.19	1.58	.114
Motivational variables												
Intrinsic value												
Mathematics	-0.14	0.17	-0.79	.428	-0.18	0.15	-1.17	.243	-0.31	0.19	-1.64	.101
Reading	0.20	0.14	1.45	.146	-0.21	0.17	-1.23	.219	-0.01	0.17	-0.03	.976
Writing	0.08	0.15	0.56	.577	0.03	0.15	0.19	.852	0.11	0.14	0.81	.417
Self-concept												
Mathematics	0.11	0.21	0.52	.603	-0.18	0.16	-1.10	.271	-0.07	0.22	-0.33	.745
Reading	0.23	0.15	1.54	.123	-0.11	0.17	-0.66	.512	0.12	0.15	0.77	.440
Writing	0.26	0.18	1.44	.149	-0.05	0.19	-0.27	.791	0.21	0.19	1.12	.262

Schools in Both Cohorts (N = 556)

Table S12. Effects of the COVID-19 Pandemic

Variables	Multiple regression coefficient (ES in SD_Y)	*SE*	*z*	*p*
Achievement				
Reading	-0.12	0.12	-1.02	.309
Mathematics	-0.01	0.12	-0.07	.948
Motivational variables				
Intrinsic value				
Mathematics	-0.06	0.12	-0.51	.612
Reading	-0.03	0.11	-0.26	.792
Writing	-0.06	0.09	-0.59	.555
Self-concept				
Mathematics	-0.09	0.08	-1.10	.271
Reading	0.04	0.10	0.41	.683
Writing	0.01	0.09	0.12	.902

Note. The table presents the regression coefficients for cohort (treatment variable) in a multiple linear regression analysis in which all dependent variables were considered simultaneously and all pretest measures were used as control variables. We ran the analysis in Mplus 8.5 (Muthén & Muthén, 1998–2017). To account for potentially nonnormally distributed variables and the multilevel structure of the data (students nested in classes), we used cluster-robust standard errors (for a justification, see McNeish et al., 2017). To handle missing values, we used full information maximum likelihood (FIML) estimation. All variables were z-standardized before the analysis except for the dichotomous variables (cohort, gender, and migration background). Thus, the regression coefficients can be interpreted as effect sizes.

Table S13. Effects of the COVID-19 Pandemic – Moderated by HISEI

Variables	Cohort effect (ES in SD_Y)				HISEI (ES in SD_Y)				Interaction term Cohort x HISEI (ES in SD_Y)			
	b	*SE*	*z*	*p*	*b*	*SE*	*z*	*p*	*b*	*SE*	*z*	*p*
Achievement												
Reading	-0.13	0.12	-1.05	.295	0.19	0.07	2.47	.014	-	-	-	-
Mathematics	|<0.01|	0.12	-0.04	.967	0.05	0.05	1.09	.277	-0.04	0.07	-0.48	.629
Motivational variables												
Intrinsic value												
Mathematics	-0.06	0.12	-0.51	.612	-0.02	0.05	-0.46	.646	-0.01	0.08	-0.18	.856
Reading	-0.03	0.11	-0.28	.779	0.11	0.08	1.26	.208	0.01	0.11	0.14	.890
Writing	-0.05	0.09	-0.50	.620	0.01	0.08	0.11	.911	-0.11	0.09	-1.14	.256
Self-concept												
Mathematics	-0.09	0.08	-1.1	.272	|<0.01|	0.06	-0.04	.969	-0.02	0.09	-0.28	.780
Reading	0.04	0.10	0.40	.689	0.13	0.05	2.62	.009	-0.01	0.1	-0.08	.939
Writing	0.02	0.09	0.24	.812	0.05	0.05	1.03	.303	-0.15	0.07	-2.04	.041

Note. The table presents the regression coefficients for cohort (treatment variable), HISEI score, and the interaction between cohort and HISEI score in a multiple linear regression analysis in which all dependent variables were considered simultaneously and all pretest measures were used as control variables. We ran the analysis in Mplus 8.5 (Muthén & Muthén, 1998–2017). To account for potentially nonnormally distributed variables and the multilevel structure of the data (students nested in classes), we used cluster-robust standard errors (for a justification, see McNeish et al., 2017). To handle missing values, we used full information maximum likelihood (FIML) estimation. All variables were z-standardized before the analysis except for the dichotomous variables (cohort, gender, and migration background). Thus, the regression coefficients can be interpreted as effect sizes.

Schools in Both Cohorts (N = 556)

Table S14. Effects of the COVID-19 Pandemic – Moderated by Baseline Level

Variables	Cohort effect (ES in SD_Y)				Pretest (ES in SD_Y)				Interaction term Cohort x Pretest (ES in SD_Y)			
	b	*SE*	*z*	*p*	*b*	*SE*	*z*	*p*	*b*	*SE*	*z*	*p*
Achievement												
Reading	-0.12	0.12	-1.00	.318	0.14	0.04	3.19	.001	-	-	-	-
Mathematics	|<0.01|	0.12	-0.02	.986	0.46	0.04	10.7	<.001	0.06	0.05	1.03	.302
Motivational variables												
Intrinsic value												
Mathematics	-0.06	0.11	-0.50	.614	0.25	0.07	3.34	.001	-0.12	0.10	-1.20	.231
Reading	-0.03	0.11	-0.27	.788	0.03	0.07	0.37	.713	0.03	0.10	0.28	.783
Writing	-0.04	0.10	-0.48	.634	0.13	0.06	2.35	.019	-0.15	0.07	-2.00	.045
Self-concept												
Mathematics	-0.09	0.08	-1.11	.266	0.12	0.07	1.84	.066	-0.06	0.09	-0.70	.484
Reading	0.04	0.10	0.41	.683	0.02	0.05	0.43	.666	0.01	0.06	0.09	.927
Writing	0.01	0.09	0.15	.879	0.07	0.07	1.08	.282	-0.05	0.08	-0.58	.563

Note. The table presents the regression coefficients for cohort (treatment variable), pretest score, and the interaction between cohort and pretest score in a multiple linear regression analysis in which all dependent variables were considered simultaneously and all pretest measures were used as control variables. We ran the analysis in Mplus 8.5 (Muthén & Muthén, 1998–2017). To account for potentially nonnormally distributed variables and the multilevel structure of the data (students nested in classes), we used cluster-robust standard errors (for a justification, see McNeish et al., 2017). To handle missing values, we used full information maximum likelihood (FIML) estimation. All variables were z-standardized before the analysis except for the dichotomous variables (cohort, gender, and migration background). Thus, the regression coefficients can be interpreted as effect sizes.

Table S15. Effects of the COVID-19 Pandemic for Different Levels of Contact Between Teachers and Students

Variables	Indicator 1 – low contact (ES in SD_Y) "occasionally, never"						Indicator 2 – medium contact (ES in SD_Y) "once a week"						Indicator 3 – high contact (ES in SD_Y) "several times per week, almost every day"					
		95% CI						95% CI										
	b	low	up	*SE*	*z*	*p*	*b*	low	up	*SE*	*z*	*p*	*b*	low	up	*SE*	*z*	*p*
Achievement																		
Reading	-0.22	-0.68	0.24	0.23	-0.95	.340	-0.32	-0.67	0.02	0.18	-1.83	.067	-0.03	-0.62	0.56	0.30	-0.10	.923
Mathematics	-0.03	-0.35	0.29	0.16	-0.18	.858	-0.14	-0.56	0.28	0.22	-0.65	.514	0.48	0.15	0.80	0.17	2.84	.005
Motivational variables																		
Intrinsic value																		
Mathematics	-0.01	-0.46	0.44	0.23	-0.04	.967	0.23	-0.20	0.67	0.22	1.05	.296	0.06	-0.68	0.81	0.38	0.17	.866
Reading	-0.09	-0.51	0.34	0.22	-0.40	.691	0.10	-0.43	0.63	0.27	0.37	.714	-0.27	-1.10	0.56	0.42	-0.63	.527
Writing	-0.17	-0.84	0.50	0.34	-0.50	.617	-0.12	-0.68	0.45	0.29	-0.41	.683	0.14	-0.37	0.65	0.26	0.54	.586
Self-concept																		
Mathematics	-0.17	-0.54	0.20	0.19	-0.90	.369	0.10	-0.31	0.51	0.21	0.46	.645	0.14	-0.42	0.70	0.29	0.49	.622
Reading	-0.07	-0.56	0.43	0.25	-0.25	.799	0.14	-0.32	0.60	0.23	0.61	.541	-0.04	-0.69	0.62	0.33	-0.10	.917
Writing	-0.08	-0.66	0.49	0.29	-0.29	.774	0.01	-0.40	0.41	0.21	0.04	.970	0.20	-0.13	0.52	0.16	1.20	.231

Schools in Both Cohorts (N = 556)

Table S16. Differences in the Effects of the COVID-19 Pandemic for Different Levels of Contact Between Teachers and Students

Variables	Difference Indicator 2 – Indicator 1 (ES in SD_Y) "once a week" vs. "occasionally or never"				Difference Indicator 3 – Indicator 2 (ES in SD_Y) "several times per week or almost every day" vs. "once a week"				Difference Indicator 3 – Indicator 1 (ES in SD_Y) "several times per week or almost every day" vs. "occasionally or never"			
	b	*SE*	*z*	*p*	*b*	*SE*	*z*	*p*	*b*	*SE*	*z*	*p*
Achievement												
Reading	-0.10	0.22	-0.46	.647	0.29	0.33	0.90	.368	0.19	0.38	0.51	.613
Mathematics	-0.11	0.22	-0.50	.615	0.62	0.25	2.51	.012	0.50	0.22	2.33	.020
Motivational variables												
Intrinsic value												
Mathematics	0.24	0.24	1.02	.308	-0.17	0.42	-0.40	.688	0.07	0.42	0.17	.862
Reading	0.19	0.28	0.65	.514	-0.37	0.48	-0.77	.440	-0.18	0.45	-0.41	.684
Writing	0.05	0.44	0.12	.904	0.26	0.36	0.73	.467	0.31	0.41	0.77	.444
Self-concept												
Mathematics	0.27	0.22	1.24	.216	0.04	0.36	0.12	.903	0.31	0.33	0.96	.339
Reading	0.21	0.26	0.79	.429	-0.18	0.38	-0.47	.638	0.03	0.41	0.07	.943
Writing	0.09	0.34	0.28	.784	0.19	0.21	0.90	.371	0.28	0.32	0.88	.381

Table S17

Descriptive Statistics (Pretest Measurement)

Variables	Entire sample (*N* = 2,740)					Cohort 1 (*N* = 1,405)				Cohort 2 (*N* = 1,335)			
	M	*SD*	Range		Rate of missing data	*M*	*SD*	Range		*M*	*SD*	Range	
			Min	Max				Min	Max			Min	Max
Achievement													
Reading T1	20.90	27.51	0	155	0.01	19.83	25.97	0	154	22.01	29.00	0	155
Reading T1 - NA	19.93	26.36	0	155	0.03	17.86	23.28	0	154	22.01	29.00	0	155
Reading T1 - Min	20.57	26.70	0	155	0.01	19.18	24.22	0	154	22.01	29.00	0	155
Reading T1 - Max	21.81	30.19	0	156	0.01	21.62	31.30	0	156	22.01	29.00	0	155
Reading T2	131.89	27.39	2	159	0.22	131.66	26.10	6	159	132.28	29.47	2	159
Reading T2 - NAs	131.41	27.49	2	159	0.26	130.86	26.13	6	159	132.28	29.47	2	159
Reading T2 - Min	127.53	31.35	2	159	0.22	124.75	32.08	6	159	132.28	29.47	2	159
Reading T2 - Max	132.80	27.35	2	159	0.22	133.10	26.03	6	159	132.28	29.47	2	159

Note. This table presents descriptive statistics for reading achievement at the beginning of Grade 1 when different ways of handling missing data—due to the programming error—were applied. Reading T1/T2 = reading score that was used in the main part of the manuscript; missing values on items were replaced by predicted values. Reading T1/T2 – NA = reading sum score with NAs instead of predicted values. Reading T1/T2 – Min = missing values on items were replaced by 0. Reading T1/T2 – Max = missing values on items were replaced by the maximum number of points.

Table S18

Baseline Differences Between Cohort 1 and Cohort 2

Variables	ES in SD_Y	*SE*	*z*	*p*
Achievement				
Reading T1	0.08	0.04	1.89	.058
Reading T1 - NA	0.14	0.04	3.30	.001
Reading T1 - Min	0.11	0.04	2.53	.011
Reading T1 - Max	0.02	0.04	0.35	.727

Note. Baseline differences are presented for reading achievement at the beginning of Grade 1 when different ways of handling missing data—due to the programming error—were applied. Reading T1 = reading score that was used in the main part of the manuscript; missing values on items were replaced by predicted values. Reading T1 – NA = reading sum score with NAs instead of predicted values. Reading T1 – Min = missing values on items were replaced by 0. Reading T1 – Max = missing values on items were replaced by the maximum number of points.

Table S19
Effects of the COVID-19 Pandemic on Reading Achievement at the End of Grade 1

Variables	Multiple regression coefficient (ES in SD_Y)	*SE*	*z*	*p*
Achievement				
Reading T1/T2	0.03	0.07	0.36	.718
Reading T1/T2 - NA	-0.01	0.07	-0.19	.851
Reading T1/T2 - Min	0.26	0.06	4.32	<.001
Reading T1/T2 - Max	-0.02	0.07	-0.33	.741

Note. Effects of the pandemic on reading achievement at the end of Grade 1 are presented for different ways of handling missing data. Reading T1/T2 = reading score that was used in the main part of the manuscript; missing values on items were replaced by predicted values. Reading T1/T2 – NA = reading sum score with NAs instead of predicted values. Reading T1/T2 – Min = missing values on items were replaced by 0. Reading T1/T2 – Max = missing values on items were replaced by the maximum number of points.

Table S20

Effects of the COVID-19 Pandemic for Different Levels of Contact Between Teachers and Students

Variables	Indicator 1 – low contact (ES in SD_Y) “occasionally, never”						Indicator 2 – medium contact (ES in SD_Y) “once a week”						Indicator 3 – high contact (ES in SD_Y) “several times per week, almost every day“					
		95% CI						95% CI										
	b	low	up	*SE*	*z*	*p*	*b*	low	up	*SE*	*z*	*p*	*b*	low	up	*SE*	*z*	*p*
Achievement																		
Reading T1/T2	-0.21	-0.50	0.09	0.15	-1.4	.163	0.06	-0.10	0.22	0.08	0.70	.481	0.14	-0.13	0.41	0.14	1.00	.317
Reading T1/T2 - NA	-0.25	-0.55	0.05	0.15	-1.65	.100	0.02	-0.14	0.18	0.08	0.26	.797	0.09	-0.19	0.37	0.14	0.66	.508
Reading T1/T2 - Min	0.08	-0.18	0.34	0.13	0.61	.544	0.28	0.13	0.43	0.08	3.64	.001	0.39	0.15	0.62	0.12	3.19	.001
Reading T1/T2 - Max	-0.26	-0.56	0.03	0.15	-1.75	.080	0.02	-0.14	0.18	0.08	0.22	.828	0.09	-0.18	0.36	0.14	0.65	.517

Note. Effects of the pandemic for different levels of contact between teachers and students on reading achievement at the end of Grade 1 are presented for different ways of handling missing data. Reading T1/T2 = reading score that was used in the main part of the manuscript; missing values on items were replaced by predicted values. Reading T1/T2 – NA = reading sum score with NAs instead of predicted values. Reading T1/T2 – Min = missing values on items were replaced by 0. Reading T1/T2 – Max = missing values on items were replaced by the maximum number of points.

Table S21
Differences between the Effects of the COVID-19 Pandemic for Different Levels of Contact Between Teachers and Students

Variables	Difference Indicator 2 – Indicator 1 (ES in SD_Y) "once a week" vs. "occasionally or never"				Difference Indicator 3 – Indicator 2 (ES in SD_Y) "several times per week or almost every day" vs. "once a week"				Difference Indicator 3 – Indicator 1 (ES in SD_Y) "several times per week or almost every day" vs. "occasionally or never"			
	b	*SE*	*z*	*p*	*b*	*SE*	*z*	*p*	*b*	*SE*	*z*	*p*
Achievement												
Reading T1/T2	0.27	0.15	1.75	.079	0.08	0.14	0.56	.573	0.35	0.20	1.77	.076
Reading T1/T2 - NA	0.27	0.15	1.80	.072	0.07	0.14	0.50	.615	0.35	0.20	1.76	.078
Reading T1/T2 - Min	0.20	0.14	1.44	.151	0.11	0.13	0.82	.410	0.31	0.17	1.76	.078
Reading T1/T2 - Max	0.28	0.15	1.84	.065	0.07	0.14	0.50	.616	0.35	0.20	1.79	.074

SI References

Forsa. (2020). Das Deutsche Schulbarometer Spezial Corona-Krise: Ergebnisse einer Befragung von Lehrerinnen und Lehrern an allgemeinbildenden Schulen im Auftrag der Robert Bosch Stiftung in Kooperation mit der ZEIT, Lehrer-Umfrage - Erstmals repräsentative, Daten zum Fernunterricht.

Huebener, M., Spieß, C. K., & Zinn, S. (2020). SchülerInnen in Corona-Zeiten: Teils deutliche Unterschiede im Zugang zu Lernmaterial nach Schultypen und -trägern. *DIW Wochenbericht*.

Immerfall, S. (2021). Unterricht und Schulen in der Pandemie: Versuch einer Zwischenbilanz. *GWP–Gesellschaft. Wirtschaft. Politik*, *70*(3), 345–356.

Initiative D21, & TU München. (2020). *Homeschooling in Zeiten von Corona - Vorabergebnisse der Studie eGovernment MONITOR 2020*

McNeish, D., Stapleton, L. M., & Silverman, R. D. (2017). On the unnecessary ubiquity of hierarchical linear modeling. *Psychological Methods*, *22*(1), 114–140. https://doi.org/10.1037/met0000078

Muthén, L. K., & Muthén, B. O. (1998–2017). *Mplus User's Guide. Eighth Edition.* Muthén & Muthén.

UNESCO. (2022). *Duration school closures*. https://en.unesco.org/sites/default/files/duration_school_closures.csv

Vodafone Stiftung Deutschland. (2020). *Schule auf Distanz: Perspektiven und Empfehlungen für den neuen Schulalltag Eine repräsentative Befragung von Lehrkräften in Deutschland*.

Wößmann, L., Freundl, V., Grewenig, E., Lergetporer, P., Werner, K., & Zierow, L. (2020). Bildung in der Coronakrise: Wie haben die Schulkinder die Zeit der Schulschließungen verbracht, und welche Bildungsmaßnahmen befürworten die Deutschen? [Education during the Corona Crisis: How have schoolchildren spent the period of school closures, and what educational programs do Germans favor?]. *ifo Schnelldienst*, *73*(9), 25–39.
